# Mutation of conserved MHC class I cytoplasmic tyrosine affects CD8+ T cell priming, effector function, and memory response

**DOI:** 10.3389/fimmu.2025.1572342

**Published:** 2025-09-30

**Authors:** Yimo Sun, Yitao Tang, Priscilla Ortiz, Barbara Nassif Rausseo, Barbara Pazdrak, Lama Elzohary, Arjun Katailiha, Amjad Talukder, Cassian Yee, Richard Eric Davis, Gregory Lizée

**Affiliations:** ^1^ UT Health Graduate School of Biomedical Sciences, University of Texas MD Anderson Cancer Center, Houston, TX, United States; ^2^ Department of Melanoma Medical Oncology, The University of Texas (UT) MD Anderson Cancer Center, Houston, TX, United States; ^3^ Department of Bioinformatics and Computational Biology, The University of Texas (UT) MD Anderson Cancer Center, Houston, TX, United States; ^4^ Department of Immunology, The University of Texas (UT) MD Anderson Cancer Center, Houston, TX, United States; ^5^ Department of Lymphoma-Myeloma, The University of Texas (UT) MD Anderson Cancer Center, Houston, TX, United States

**Keywords:** MHC class I, human leukocyte antigen (HLA), cytotoxic T cells, dendritic cells, tyrosine phosphorylation, cytoplasmic tail

## Abstract

The cytoplasmic domain of MHC class I (MHC-I) molecules contains a single, highly conserved tyrosine residue (Y320). In previous work, we found that mice expressing a Y320F-mutated form of H-2K^b^ had reduced capacity to generate K^b^-restricted cytotoxic T lymphocyte (CTL) responses following viral infection, due at least in part to defects in endolysosomal trafficking of H-2K^b^ and antigen cross-presentation by dendritic cells (DCs). In this study, we investigated whether there are additional, post-presentation dependencies on Y320 for T cell priming. We engineered both human- and mouse-derived antigen-presenting cells (APCs) to express either wild-type MHC-I or variants of MHC-I containing Y320F or Y320E mutations. We found that Y320E-mutated HLA-A*0201 elicited enhanced *in vitro* priming and expansion of human antigen-specific CD8+ T cells, which showed a unique transcriptional profile compared to T cells primed with APCs expressing either WT or Y320F-mutated A*0201. Furthermore, the Y320E variant of H-2K^b^ expressed in the context of a murine DC vaccine model induced altered T cell differentiation kinetics while improving both anti-tumor immunity and augmenting the magnitude of memory CD8+ T cell responses *in vivo*. These results suggest that Y320 phosphorylation of MHC-I may play a role in determining the fate and function of CD8+ T cells and suggest a novel strategy for improving DC-based cancer immunotherapies.

## Introduction

The short cytoplasmic domains of classical MHC-I molecules (~35 amino acids) display striking evolutionary conservation at three sites of potential phosphorylation: two serine residues (S332 and S335) encoded by exon 7, and a single tyrosine residue (Y320) encoded by exon 6 (1–6). Serine phosphorylation of the MHC-I cytoplasmic tail has been associated with oncogenic signaling, internalization, and intracellular trafficking, although the kinases involved have yet to be identified, and exon 7-deleted forms of MHC-I have been shown to elicit superior CD8+ T cell responses in both mouse and human studies (1, 7–11). Although the serine kinases targeting the MHC-I tail have yet to be identified definitively, phosphorylation of Y320 (pY320) by Src kinase has been demonstrated *in vitro*, and mass spectrometry studies of various cell types have found pY320-containing peptides (3, 7, 12–14). Functional studies involving tyrosine phosphorylation of MHC-I have largely involved cell types other than APCs (4), but tyrosine phosphorylation of MHC-I was induced by Toll-like receptor (TLR) ligand-mediated activation of mouse macrophages, suggesting a link between pathogenic inflammation and the induction of MHC-I tyrosine phosphorylation in APCs (15). Intriguingly, Y320 is also a direct target of the HIV-*nef* protein, through which it can drive the downregulation of surface HLA-A and -B to promote immune evasion during HIV infection (16–19).

Previous studies showed that transgenic mice expressing H-2D^b^ containing a glycophosphatidylinositol (GPI)-lipid anchor in place of the transmembrane and cytoplasmic domains demonstrated significantly diminished D^b^-specific CTL responses against an immunodominant Influenza A epitope. Subsequent work demonstrated that mice expressing Y320F point-mutated H2-K^b^ were similarly impaired in their capacity to generate immunodominant K^b^-restricted CTL responses following vesicular stomatitis virus (VSV) or Sendai virus infection. Bone marrow-derived DCs from these Y320F mice were also impaired in their ability to cross-present the K^b^-restricted OVA peptide following exposure to exogenous ovalbumin protein. Furthermore, Y320F-mutated K^b^ molecules showed intracellular trafficking defects that rendered them incapable of traversing through endolysosomal compartments of DCs that are the primary sites of OVA peptide loading for WT-K^b^ molecules, consistent with other reports (1, 20, 21). It is believed that the conserved MHC-I cytoplasmic YXXA sequence motif is analogous to tyrosine-based endocytic sorting signals with a YXXØ motif (where Ø is a large hydrophobic amino acid) that are found in many other transmembrane proteins (2, 22).

Although incapable of cross-presentation, Y320F-mutated K^b^ molecules were shown to be fully capable of being peptide-loaded and presented at the cell surface through the conventional antigen presentation pathway or peptide pulsing (1). It is therefore possible that at least some of the defect in antiviral CTL responses observed in Y320F-K^b^ mice may be intrinsic to MHC-I function, occurring despite peptide loading and surface presentation. In this study, we sought to investigate the post-presentation role of MHC-I Y320 phosphorylation in determining the fate and effector function of CD8+ T cells. In the absence of a specific way to influence the phosphorylation state of MHC-I Y320, we engineered human or mouse APCs to express HLA-A*0201 or H-2K^b^ molecules bearing glutamic acid at position 320 (Y320E), previously used to mimic the biological activity of phosphorylated tyrosine in other proteins (23, 24). As before, Y320F mutants of A*0201 or H-2K^b^ were expressed in APCs to mimic unphosphorylated tyrosine. In addition, we employed “single-chain trimer” (SCT) molecules that covalently link the MHC-I heavy chain, β2-microglobulin, and antigenic peptide (25), in part because SCTs are not subject to cross-presentation (26). Utilizing these engineered APCs, we found that the Y320E variants not only facilitated enhanced priming and expansion of CD8+ T cells against multiple epitopes *in vitro*, but also promoted superior antitumor immunity and CD8+ T cell memory in the context of a DC vaccine *in vivo*.

## Results

### Y320E mutation of HLA-A*0201 enhanced antigen-specific CD8+ T cell priming and expansion *in vitro*

**Figure 1:**
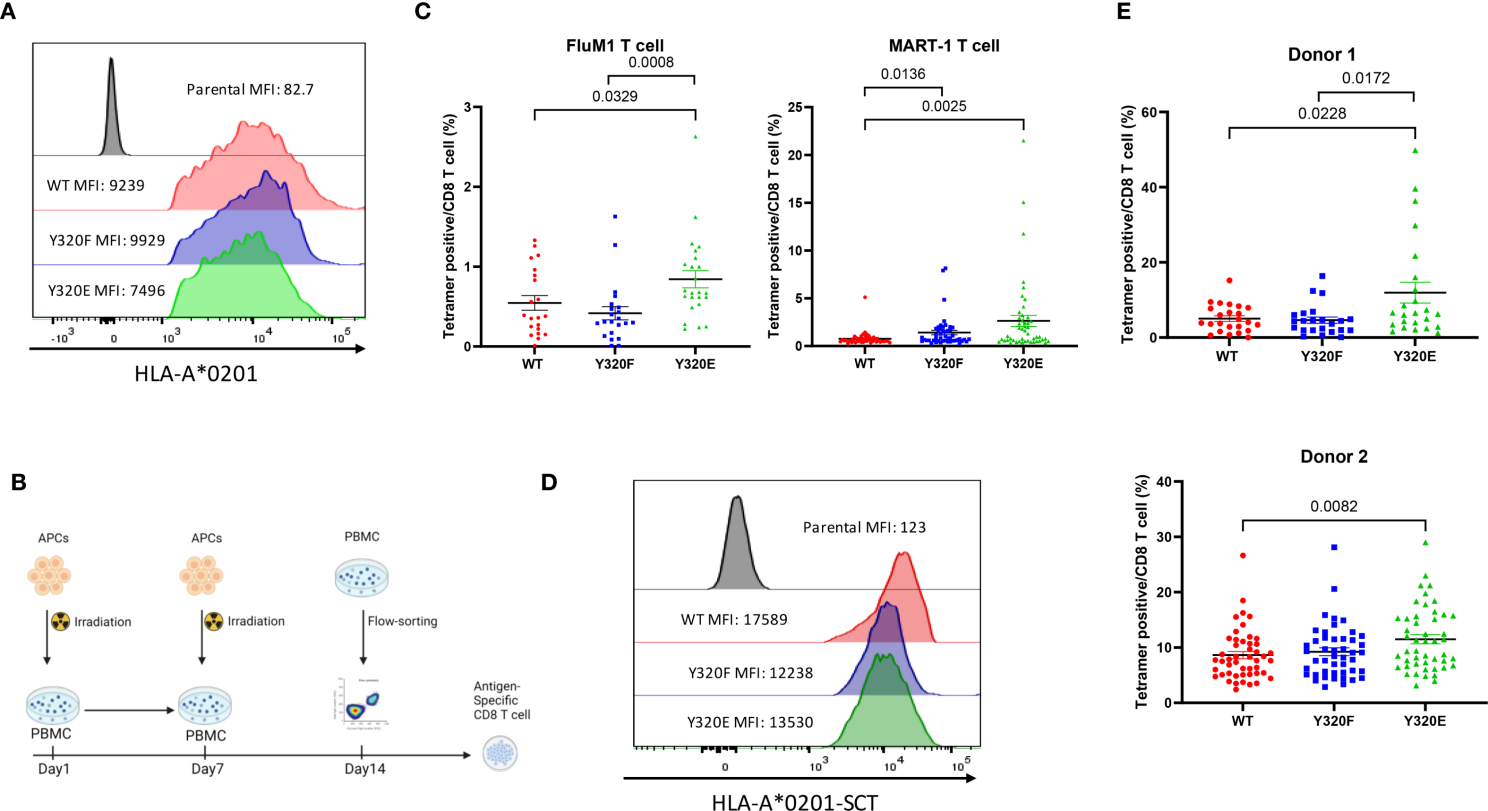
Y320E mutation enhances human T cell priming in vitro. **(A)** Surface expression of HLA A*0201 forms on transduced KG-1 cells assessed by flow cytometry. **(B)** Schematic representation of the workflow for antigen-specific CD8^+^ T cell priming using healthy donor PBMCs in vitro. **(C)** Frequency of antigen-specific T cells after in vitro priming with pulsed FluM1 and MART-1 peptides in two different donors. Statistical differences among groups were determined using one-way ANOVA (FluM1: p = 0.0067; MART-1: p = 0.0015), followed by pairwise comparisons using an unpaired two-tailed t-test (only significant p values are shown). **(D)** Surface expression of OVA peptide-bearing HLA-A*0201 single-chain trimers on transduced KG-1 cells assessed by flow cytometry. **(E)** Comparison of FluM1 antigen-specific T cell frequencies in two donors primed with KG-1 cells transduced with single-chain trimer (SCT) constructs expressing FluM1 antigen. Donor 1 is the same as was used for FluM1 peptide in panel **(C)** Group differences were analyzed using one-way ANOVA (Donor 1: p = 0.0055; Donor 2: p = 0.0164), with pairwise comparisons performed using an unpaired two-tailed t-test. All data are presented as mean ± standard error of the mean (SEM). Statistically significant p values (p < 0.05) are indicated in the graph. For Donor 1, the experiment was repeated three times, and for Donor 2, it was repeated twice to ensure reproducibility.

To study priming of human CD8+ T cells, human macrophage-derived KG-1 cells (which lack endogenous HLA-A*0201 expression) were transduced with lentiviral vectors encoding wild-type (WT) A*0201 or cytoplasmic tail-mutated Y320F or Y320E variants to serve as APCs. Flow cytometry of the engineered KG-1 cells confirmed that the A*0201 variants displayed similar levels of transcription and cell surface expression (**Figures 1A**, **Supplementary Figure S1A**). Engineered KG-1 cells were pulsed with the A*0201-restricted FluM1 peptide GILGFVFTL, irradiated, and cocultured with peripheral blood mononuclear cells (PBMCs) obtained from healthy A*0201-positive human donors for two weekly stimulations (**Figure 1B**). Expanded FluM1-specific CD8+ T cells were then quantitated via flow cytometry using anti-CD8+ and a FluM1/A*0201 tetramer, revealing that a significantly higher proportion of FluM1-specific T cells was elicited by the Y320E-mutated A*0201, compared to either the WT or Y320F-mutated forms of A*0201 (**Figure 1C**). FluM1-specific T cells in human PBMC largely consist of effector memory cells generated by prior influenza exposure; in contrast, normal donors of HLA-A*0201 genotype have substantial numbers of CD8+ T cells recognizing the MART-1 melanocyte peptide antigen AAGIGILTV, and these are predominantly naïve (27). As a test of *in vitro* priming of naïve CD8+ T cells, we incubated PBMCs from HLA-A*0201 donors with engineered KG-1 cells pulsed with the MART-1 peptide. Consistent with the FluM1 results, Y320E-mutated A*0201 significantly increased priming and expansion of MART-1-specific CD8+ T cells compared to WT-A*0201 (**Figure 1C**).

Since pulsed peptides are not covalently linked to MHC-I complexes, it was possible that they might dissociate from the KG-1 cells for subsequent binding and presentation by endogenous WT-A*0201 molecules that are highly expressed on PBMC of A*0201-positive donors. To control for this, we employed A*0201 “single-chain trimer” (SCT) molecules that effectively mimic natural HLA-I/peptide complexes generated via conventional antigen processing (28–30) (**Supplementary Figure S1B**). We transduced KG-1 cells to express WT or cytoplasmic tail mutant (Y320F or Y320E) forms of A*0201/FluM1 SCTs, producing similar cell surface levels (**Figure 1D**), and repeated the stimulation experiment with PBMC from two additional healthy A*0201-positive donors. The results were similar to those observed using peptide-pulsed KG-1 cells, with the Y320E-mutated A*0201-SCT eliciting significantly higher levels of FluM1-specific CD8+ T cell expansion compared to the WT A*0201-SCT (**Figure 1E**).

### Y320E-primed antigen-specific human T cells showed a distinct transcriptional profile compared with A*0201(WT)-primed T cells

**Figure 2:**
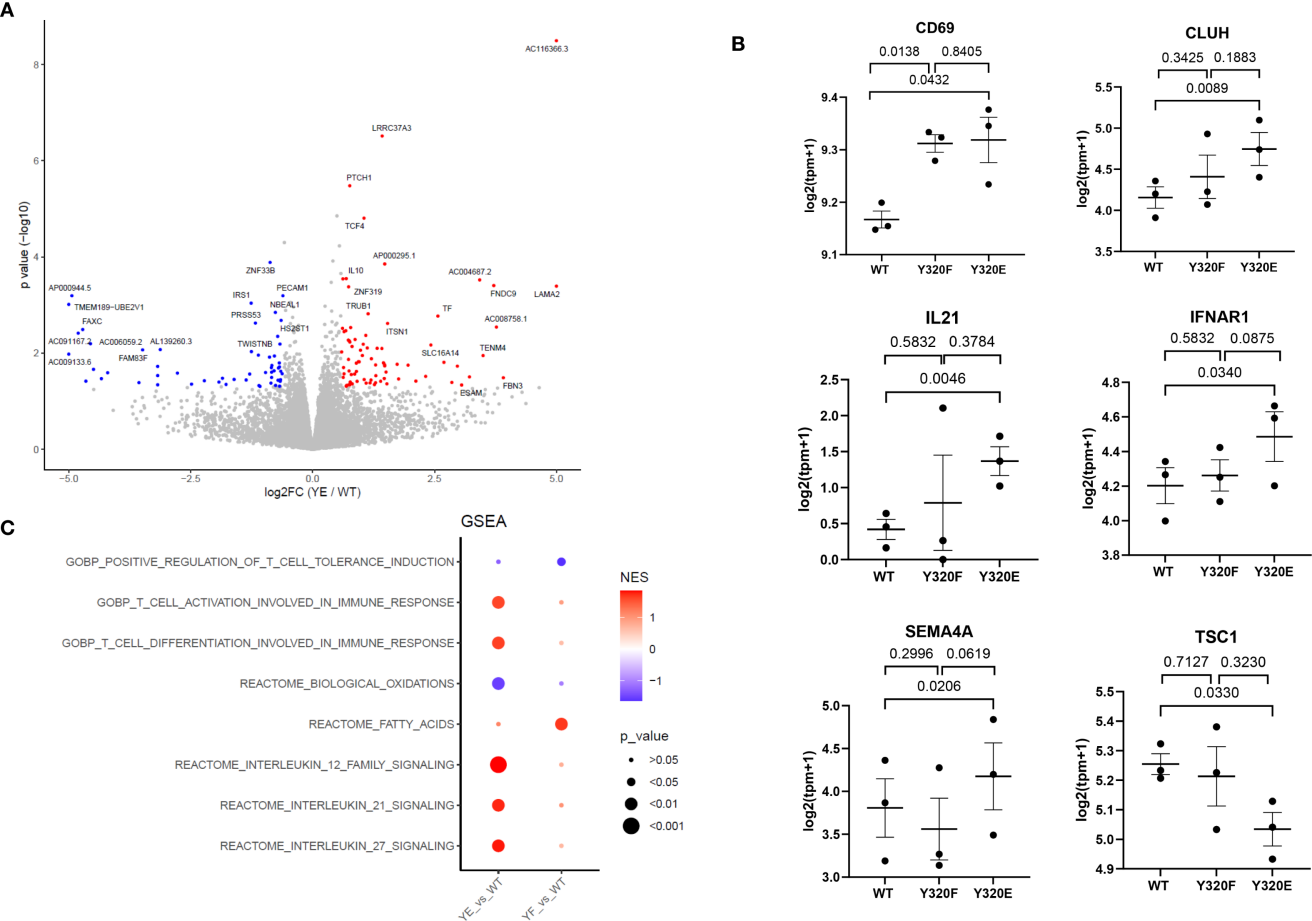
Transcriptomic analysis of human primed antigen-specific CD8+ T cells reveals effects of HLA-A*0201 Y320 mutations. **(A)** Volcano plot displaying differentially expressed genes between Y320E-primed and WT-primed T cells. PBMC from 3 healthy donors were stimulated twice with KG-1 cells expressing WT or Y320E SCTs bearing FluM1 peptide, then CD8+/tetramer+ cells were sorted for RNA-Seq. Statistical significance was determined using an unpaired two-tailed Student’s t-test with Benjamini-Hochberg correction for multiple comparisons. **(B)** Expression levels of selected genes in WT-, Y320F-, and Y320E-primed T cells. Each data point represents the value for an individual donor, with paired comparisons performed using a two-tailed paired Student’s t-test. Data are presented as mean ± standard error of the mean (SEM) from n = 3 donors. **(C)** Gene Set Enrichment Analysis (GSEA) of differentially expressed genes, highlighting enriched pathways in Y320E- or Y320F-primed T cells relative to WT-primed T cells. Enrichment scores were calculated using the Kolmogorov-Smirnov statistic.

To determine whether CD8+ T cells primed by the different A*0201 SCT variants exhibited any transcriptomic differences, we performed the same KG-1 stimulation protocol using three independent, healthy PBMC donors. Following two rounds of PBMC stimulation with KG-1 cells bearing WT, Y320E-, or Y320F-mutated SCTs, antigen-specific T cells were isolated using FluM1 peptide-specific tetramers and bulk RNA sequencing (RNA-seq) was conducted. RNA-seq analysis revealed that Y320E SCT-primed T cells showed a distinct transcriptomic signature (**Figure 2A**), with upregulation of genes related to T cell activation, differentiation, and effector functions, including CD69, CLUH, IL21, and IFNAR1 (**Figure 2B**, **Supplementary Figure S2**). Notably, SEMA4A transcript expression was also upregulated (**Figure 2B**), consistent with its established role in enhancing CD8+ T cell activation and differentiation. Previous studies have identified SEMA4A as a key mediator of effector function and proliferation in antigen-specific CD8+ T cells within tumor microenvironments (TMEs) of both humans and mice (31, 32). We also observed significant downregulation of the TSC1 gene within FluM1 tetramer-positive T cells expanded with the Y320E A*0201 variant (**Figure 2B**). TSC1 is associated with maintaining cellular quiescence in CD8+ T cells, and reduced TSC1 expression aligns with increased activation and proliferative potential, further indicating that Y320E-primed T cells exhibit an effector-like phenotype (33).

These individual RNA-seq findings suggested that Y320E SCT-primed T cells exhibit a heightened activation state. Furthermore, Gene Set Enrichment Analysis (GSEA) revealed enrichment of pathways associated with T cell activation and differentiation, while pathways associated with oxidative phosphorylation were downregulated (**Figure 2C**). A metabolic shift characterized by reduced reliance on oxidative phosphorylation is consistent with the transition from a resting state to an activated effector state in CD8+ T cells (31, 34, 35). Taken together, these findings provide supportive evidence that T cells stimulated by A*0201 molecules containing a cytoplasmic Y320E mutation acquire a distinct transcriptomic profile, potentially indicative of heightened activation, differentiation, and effector potential.

### Dendritic cell vaccination with Y320E-mutated H-2Kb altered the dynamics and phenotype of primed CD8+ T cells in peripheral blood

To assess how mutations to Y320 of MHC-I molecules may impact CD8+ T cell priming, effector function, and phenotype *in vivo*, SCT complexes linking the H2-K^b^ heavy chain, β2m, and OVA peptide (SIINFEKL) were constructed (**Supplementary Figure S3A**) (25). We expressed WT-K^b^, Y320F-K^b^, or Y320E-K^b^ SCT variants in the syngeneic DC2.4 cell line, which has been used extensively for murine DC vaccination and is capable of eliciting robust anti-viral and anti-tumor responses both *in vitro* and *in vivo* (36–39). Flow cytometric analysis using an antibody specific for K^b^/OVA complexes demonstrated similar levels of cell surface Kb/OVA peptide presentation by WT, Y320F, or Y320E SCT variants on sorted DC2.4 cells (**Supplementary Figure S3A**). Furthermore, using flow cytometric analysis and confocal microscopy (**Supplementary Figures S3B, C**, respectively), we found no apparent difference in rates of internalization between the different SCT variants.

**Figure 3:**
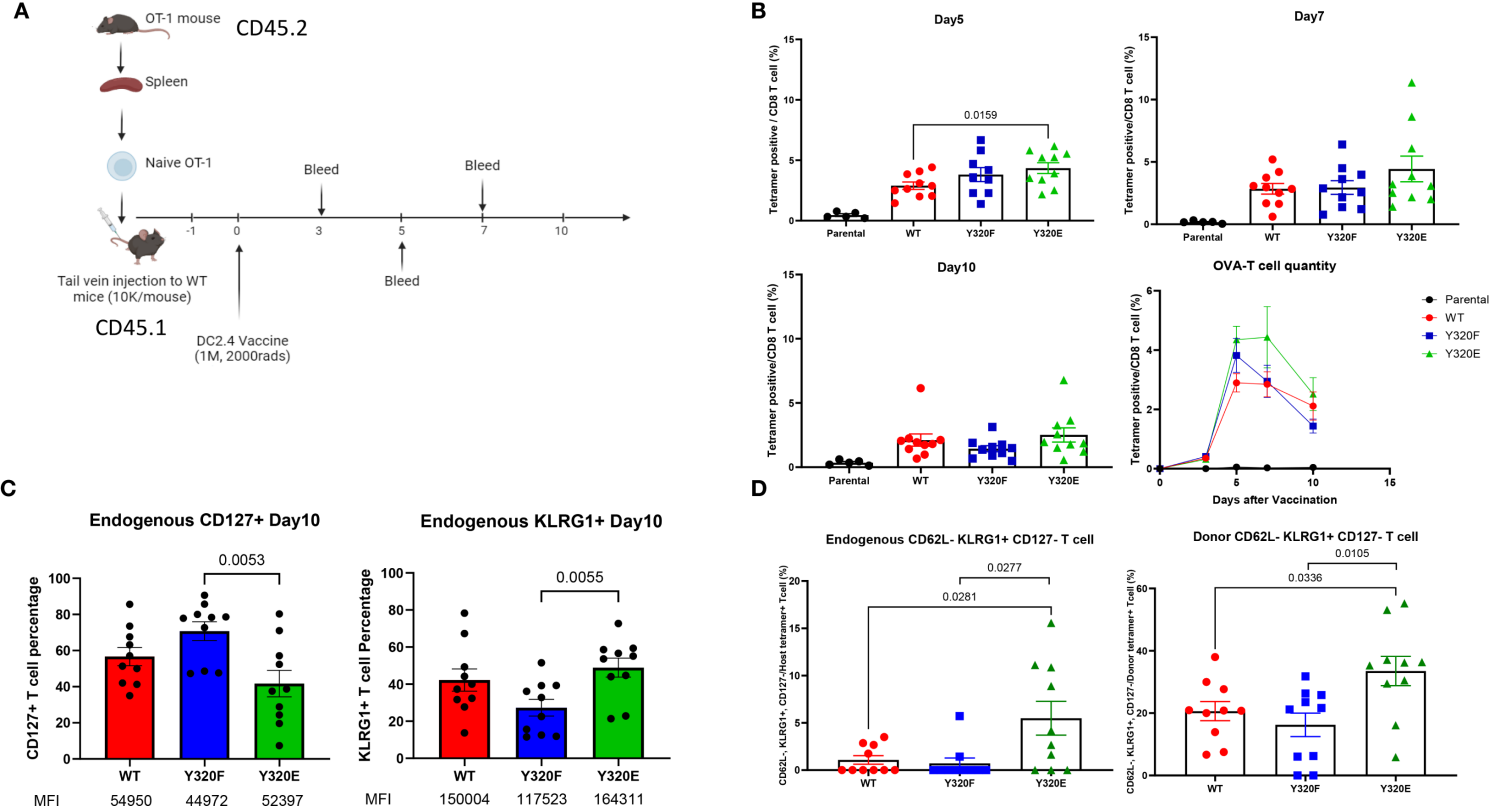
Y320E mutation enhances CD8+ T cell priming in vivo. **(A)** Schematic representation of the OT-1 T cell transfer and DC/SCT vaccination strategy. **(B)** Relative frequency of OVA-specific T cells in peripheral blood at multiple time points post-DC vaccination. Statistical differences across time points were assessed using repeated measures one-way ANOVA (p < 0.0001), followed by pairwise comparisons using an unpaired two-tailed t-test; only significant p values (p < 0.05) are shown. On day 5, WT vs. Y320F: p = 0.1653; on day 7, WT vs. Y320E: p = 0.1719. **(C)** Percentage of CD127+ and KLRG1+ cells within endogenous OVA specific T cells 10 days post-DC vaccination. Statistical differences between groups were analyzed using one-way ANOVA (CD127^+^: p = 0.0073; KLRG1^+^: p = 0.0288), with pairwise comparisons performed using an unpaired t-test; only significant p values are shown. The median fluorescence intensity (MFI) of positive cells is also shown beneath the labels. **(D)** Frequency of CD44^+^ CD62L^-^ KLRG1^+^ CD127^-^ OVA-specific T cell populations across different DC-vaccinated groups on day 10 post-vaccination. Statistical differences between groups were assessed using one-way ANOVA (Host: p = 0.0088; Donor: p = 0.0114), with pairwise comparisons performed using an unpaired t-test; only significant p values are shown. Data are presented as mean ± standard error of the mean (SEM).

To control for variation in the number of OVA-specific precursors in the T cell populations of WT mice, naïve OT-I T cells from CD45.2 donor mice were adoptively transferred into CD45.1 recipient mice 24 hours prior to vaccination with irradiated DC2.4 cells expressing the K^b^-SCT variants (**Figure 3A**). Five days post-immunization, we observed that Y320E-SCT vaccination induced a significantly higher frequency of OVA-specific T cells in peripheral blood, compared to either WT- or Y320F-SCT (**Figure 3B**), consistent with our human *in vitro* studies. Co-staining for CD45.1 and CD45.2 allowed for discrimination between endogenous and adoptively-transferred OVA-specific T cells, which unexpectedly revealed distinct patterns of differentiation for the two populations. Interestingly, the frequency of endogenous CD127+ OVA-specific T cells was lower on Day 10 after Y320E-SCT vaccination than with WT- or Y320F-SCT vaccination, possibly indicating a delay in memory cell formation, although the difference was only significant between Y320E and Y320F (**Figure 3C**). CD127+ frequency in endogenous OVA-specific T cells was also lower on Days 5 and 7 after Y320E-SCT vaccination, and significantly different from both WT- and Y320F-SCT vaccination (**Supplementary Figure S4A**). Conversely, Y320E-SCT vaccinated mice exhibited a higher percentage of KLRG1-positive OVA-specific endogenous T cells compared to the other 2 groups at these same time points, potentially indicative of a more highly differentiated effector state, with similar variation in the significance of comparisons (**Figures 3C**, **Supplementary Figure S4B**). Interestingly, these differences in T cell phenotype and kinetics were not observed in adoptively-transferred OT-I T cells analyzed from the same vaccinated mice (**Supplementary Figures S4A, 4B**).

On day 10 post-immunization, OVA-specific T cells from both endogenous and transferred OT-I populations primed from Y320E-SCT vaccinated mice contained a significantly higher proportion of cells with a CD44^+^CD62L^-^CD127^low^KLRG1^high^ phenotype, indicating a highly differentiated effector T cell state (**Figure 3D**). These results demonstrate that stimulation with APCs expressing Y320E-mutated K^b^ can drive a sustained expansion and differentiation of effector CD8+ T cells that is distinct from stimulation with WT- or Y320F-K^b^. The altered differentiation status and memory T cell formation kinetics observed with Y320E suggests that this phosphomimetic mutation to MHC-I can drive CD8+ T cells toward a more potent and sustained effector profile.

To determine whether these differences in priming were tissue-specific, we also assessed the phenotypes of vaccine-induced T cells in the spleen. WT C57BL/6 mice were immunized with irradiated DC2.4 cells expressing the respective SCT constructs (1 × 10^6 cells/mouse, s.c. flank), and splenocytes were harvested on day 7 for flow cytometric analysis. T cell subsets were defined as Teff (CD62L^-^, KLRG1^+^, CD127^lo^, CX3CR1^hi^), Tem (CD62L^-^, KLRG1^-^, CD127^lo^, CX3CR1^lo^), and Tcm (CD62L^+^, KLRG1^-^, CD127^hi^, CXCR3^hi^). In contrast to the peripheral blood findings described above, no significant differences in splenic CD8+ T cells were observed between SCT variants (**Supplementary Figure S5**). This discrepancy may be explained by tissue-specific factors: peripheral blood assessments may better capture recent immune activity near the vaccination site, whereas the spleen reflects distinct kinetics of T cell differentiation and maintenance (40, 41). Alternatively, this may indicate tissue-specific differences in priming with DC2.4-expressed SCTs, which do not undergo cross-presentation; it may also reflect the absence of a substantial population of naïve, high-affinity CD8+ T cells, since priming in the context of adoptive transfer of OT-I cells was not assessed in this experiment (42, 43).

### CD8+ T cells initially primed with Y320E-mutated H-2K^b^ demonstrated faster memory recall responses upon boosting

**Figure 4:**
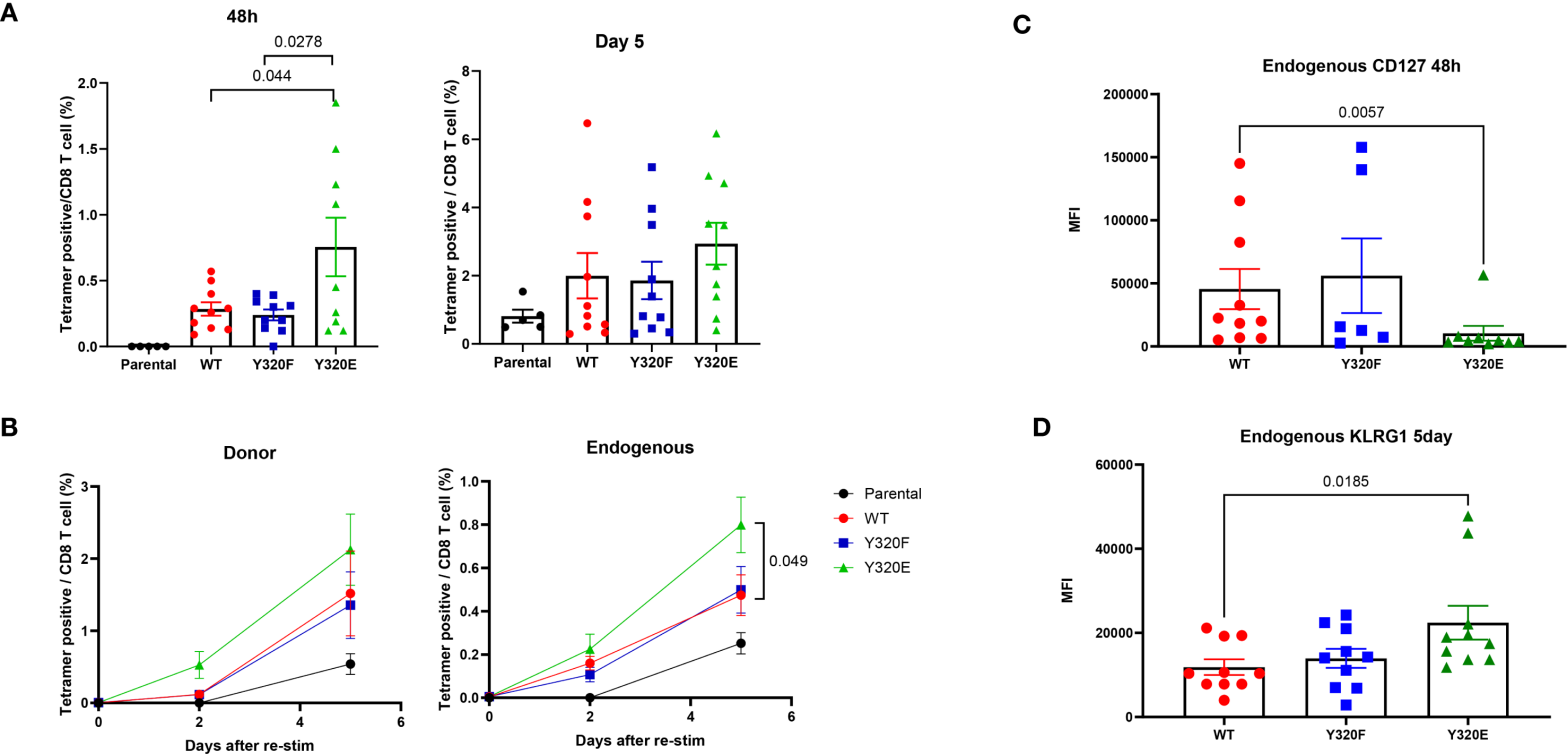
Y320E mutation in DC2.4/SCT vaccination improves memory T cell response to rechallenge. **(A)** Relative frequency of OVA-specific T cells in peripheral blood 48 hours and 5 days post-restimulation with WT DC2.4-OVA-SCT across different vaccinated groups. Statistical differences between groups were assessed using one-way ANOVA (48 hours: p = 0.0032; 5 days: p = 0.2048), followed by pairwise comparisons at 5 days using an unpaired t-test; only significant p values (p < 0.05) are shown. **(B)** Relative frequency of endogenous and donor OVA-specific T cells in peripheral blood post-restimulation with WT DC2.4-OVA-SCT across different vaccinated groups at various time points. Statistical differences between groups were assessed using one-way ANOVA, followed by pairwise comparisons using an unpaired t-test; only significant p values (p < 0.05) are shown. **(C)** Expression of CD127, shown as the median fluorescence intensity (MFI), of peripheral OVA-specific T cells 48 hours post-restimulation. Statistical differences between groups were analyzed by the Kruskal-Wallis test (p = 0.0234), followed by pairwise comparisons using an unpaired Mann-Whitney test; only significant p values (p < 0.05) are shown. **(D)** KLRG1 expression (MFI) on peripheral OVA-specific T cells 5 days post-restimulation. Statistical differences between groups were analyzed by the Kruskal-Wallis test (p = 0. 0352), followed by pairwise comparisons using an unpaired Mann-Whitney test; only significant p values (p < 0.05) are shown. Data are presented as mean ± SEM.

Since the previous results suggested that vaccinating with APCs expressing Y320E-mutated MHC-I may influence memory T cell differentiation kinetics, we next asked whether Y320E-expressing APCs can impact memory CD8+ T cell responses upon re-exposure to antigen. To answer this question, we adoptively transferred OT-I naïve T cells into recipient mice, followed by vaccination with DC2.4 cells engineered to express WT-, Y320F-, or Y320E-SCTs. Thirty days later, all vaccinated mice were given a booster vaccine consisting of DC2.4 cells expressing a WT K^b^/OVA single-chain trimer. Immediately prior to the boost, OVA-specific T cells were barely detectable across all groups. However, 48 hours after the second immunization, mice in the Y320E-SCT vaccinated group exhibited a significantly higher frequency of peripheral blood OVA-specific T cells compared to mice in the WT and Y320F groups (**Figure 4A**). Both transferred OT-I and endogenous OVA-specific T cells showed a trend of more robust expansion in the Y320E-SCT group following the secondary antigen encounter, with endogenous OVA-specific T cells expanding significantly faster in the Y320E-SCT group (**Figure 4B**). This indicates a faster and more efficient memory response. Additionally, 48 hours after the second vaccination, endogenous OVA-specific T cells in the Y320E-SCT group expressed significantly lower surface CD127 levels, indicative of heightened activation status (**Figure 4C**). Five days after the re-immunization, these cells exhibited elevated KLRG1 expression, consistent with a highly differentiated phenotype (**Figure 4D**). These findings suggested that initial vaccination with Y320E-expressing APCs promoted a more rapid and robust memory T cell response upon antigen re-encounter.

### DC vaccination with Y320E-mutated H-2K^b^ elicited better tumor control and altered effector-to-memory T cell ratios

**Figure 5:**
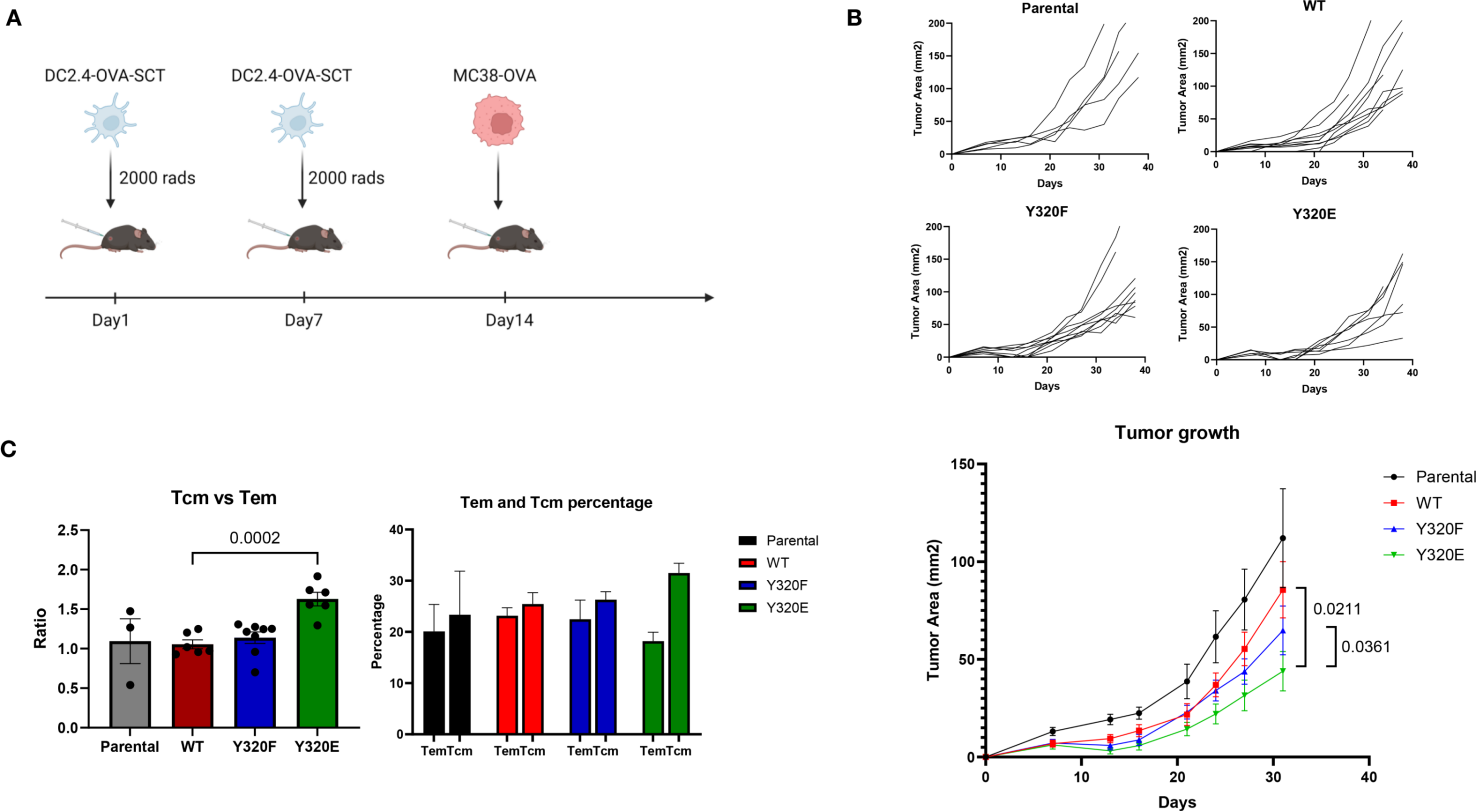
Y320E mutation in DC2.4/SCT vaccination enhances tumor control. **(A)** Schematic representation of the tumor control study. **(B)** Tumor growth curves for individual mice and combined tumor growth data across groups vaccinated with different DC2.4-OVA-SCT constructs. In the parental, WT, and Y320F groups, all mice developed tumors, whereas in the Y320E group, one mouse remained tumor-free. Tumor growth data are presented as mean ± standard error of the mean (SEM) until day 32, after which some mice were sacrificed due to tumor ulceration or tumor volume exceeding 150 mm². Statistical comparisons of tumor growth trajectories between groups were conducted using a repeated measures two-way ANOVA, followed by pairwise comparisons using an unpaired t-test for daily measurements. Statistically significant p values (p < 0.05) are indicated in the figure. WT vs. Y320F: p = 0.0882. **(C)** Ratio and percentages of central memory T cells (Tcm) and effector memory T cells (Tem) in CD8+ cells from tumor-draining lymph nodes of mice vaccinated with different DC2.4 constructs. Statistical differences between groups were assessed using one-way ANOVA (p < 0.0001). Pairwise comparisons between groups were conducted using an unpaired t-test; only significant p values (p < 0.05) are shown. Data are presented as mean ± SEM.

Building on the observation that Y320E-mutated MHC-I altered the differentiation of antigen-specific effector T cell populations and influenced memory T cell development, we next sought to determine whether these features translated into superior anti-tumor immunity *in vivo*. To this end, C57BL/6 mice were immunized twice with irradiated parental DC2.4 cells or DC2.4 cells expressing WT-, Y320F-, or Y320E-OVA-SCT variants via subcutaneous injection, followed by challenge with syngeneic MC38-OVA colon carcinoma cells (**Figure 5A**). Five weeks following tumor inoculation, mice that received the Y320E-SCT vaccine demonstrated significantly delayed MC38-OVA tumor growth compared with the other 3 groups of vaccinated mice (**Figure 5B**).

To investigate the underlying mechanisms of this enhanced tumor control, we analyzed tumor-infiltrating lymphocytes (TILs) and tumor-draining lymph nodes (TLNs) 38 days after MC38-OVA cell inoculation. Flow cytometric analysis of CD8+ TILs showed no clear differences in CD25, CD69, PD-1, CTLA-4, IFN-gamma, or perforin expression between vaccination groups (**Supplementary Figure S6**). By contrast, analysis of TLNs revealed a distinct difference in memory T cell distribution between different groups of immunized mice. Although the overall percentage of CD8+ T cells in TLNs remained consistent across all groups, Y320E-vaccinated mice exhibited a significantly higher ratio of CD62L^high^CD44^high^ cells, which we interpret as central memory T cells (Tcm), to CD62L^low^CD44^high^ cells, which we interpret as effector memory T cells (Tem; **Figure 5C**). These findings suggested that Y320E vaccination promoted enhanced central memory T cell distribution in TLNs, which may have contributed to sustained anti-tumor immunity. Collectively, these results suggest that Y320E vaccination may promote improved anti-tumor immunity through priming both highly differentiated effector and robust central memory CD8+ T cell populations.

## Discussion

In this study, we sought to advance our current understanding of the role of the MHC-I cytoplasmic domain, and particularly that of Y320, in the priming of CD8+ T cell responses. The results are consistent with previous studies showing that MHC-I cytoplasmic tail mutations can impact antiviral CTL responses *in vivo* (1, 2), and complement those earlier findings by exploring the use of Y320E-mutated forms of human and mouse MHC-I. Since Y320 has also been implicated in facilitating MHC-I molecular trafficking to endolysosomal peptide-loading compartments of APCs and antigen cross-presentation (20), we employed single-chain trimer (SCT) constructs to bypass both the conventional and cross-presentation pathways (25). This approach enabled direct evaluation of how Y320 mutations affect CD8+ T cell priming when antigen is presented as a pre-assembled MHC-I/peptide complex.

*In vitro*, Y320E mutations to A*0201 enhanced human CTL priming and expansion, yielding a higher frequency of antigen-specific T cells with increased activation status. *In vivo*, vaccination with DC2.4 cells expressing Y320E-mutated H-2K^b^/OVA SCTs similarly promoted sustained expansion of effector T cells and altered differentiation dynamics in peripheral blood. The Y320E mutation also accelerated memory recall responses and enhanced protection against tumor challenge. Together, these results support a model in which phosphorylation at Y320 promotes more effective antigen-specific CD8+ T cell responses by enhancing both the magnitude and the quality of effector and memory CTL differentiation.

The current study has several limitations which advise that results be interpreted with some caution. First and foremost, while tyrosine-to-glutamic acid point substitutions can effectively mimic phosphotyrosine (pY) in some settings (23, 24), this has not been formally demonstrated in the context of MHC-I. It is thus possible that Y320E mutations to MHC-I only weakly mimic the effect of Y320 phosphorylation, since glutamate lacks an aromatic structure and possesses a significantly weaker negative charge. This could imply that phosphorylated Y320 may have an even stronger impact on CD8+ T cell priming than observed with the phosphomimetic mutation, although this remains to be demonstrated. A further limitation of this study is that although the model systems used are well-established, they do not fully represent natural immune responses in which physiological antigen densities, numbers of responding CD8+ T cell precursors, and APC-cognate T cell interactions are lower by several orders of magnitude. While these models have clear drawbacks, it is also possible that the optimized conditions may have served to mask potentially stronger impacts of the Y320 mutations, which may become apparent when these parameters are naturally limited. Finally, our current lack of ability to monitor the pY320 status of MHC-I in either human or mouse cells represents a severe limitation to directly assessing the impact of this tyrosine phosphorylation event. Overcoming this technical challenge by developing specific tools to enable detection and quantitation of pY320 will be crucial for advancing our understanding of this potentially important modification.

Although the Y320E-mutated forms of MHC-I generally elicited more robust and persistent CD8+ T cell responses in our study, Y320F-mutated MHC-I curiously did not consistently show significant differences in priming compared to WT MHC-I (1); in most experiments, WT and Y320F-mutated MHC-I molecules elicited similar results that were distinct from Y320E. The simplest explanation for this observation is that in the APCs employed in our studies, Y320 phosphorylation either does not occur naturally or is strongly inhibited; consistent with this, mass spectrometry-based analysis of WT A*0201 molecules in KG-1 cells failed to detect Y320 phosphorylation (unpublished data not shown). If Y320 phosphorylation is indeed a signal for promoting CD8+ T cell responses, it might be expected to be downregulated in tumor cells, including KG-1 and DC2.4 cells. However, in some experiments Y320F-mutated MHC-I elicited a phenotype intermediate between WT and Y320E. Based on this, we speculate that the Y320F mutation may affect MHC-I function in additional ways beyond a lack of phosphorylation; for example, Y320F mutations to MHC-I have previously been shown to affect complex stability at the APC surface, which could conceivably alter antigen presentation to T cells compared to WT MHC-I (20, 21). Phenylalanine itself differs significantly from tyrosine in terms of hydrophobicity, which could impact the degree to which residue 320 is exposed or potentially alter the natural structure of the MHC-I tail. Further mechanistic studies will be required to distinguish between these possibilities.

The mammalian tyrosine kinases and phosphatases that may act on Y320 remain largely unknown, although Rous sarcoma virus kinase (*pp60v-src*) was previously demonstrated to facilitate Y320 phosphorylation of HLA-A and -B molecules *in vitro*, and a Src-family tyrosine kinase inhibitor prevented tyrosine phosphorylation of MHC-I (4, 7). A more recent study reported that phosphorylation of Y320 of MHC-I could be induced in primary macrophages by TLR-mediated inflammatory signals (15), an observation that dovetails nicely with the results of the current study, in which Y320E consistently promoted the augmentation of CTL responses. A major mechanism by which HIV causes immunodeficiency *in vivo* is through HIV-*nef*, a viral protein that specifically binds to Y320 to downregulate the surface expression of HLA-A and -B molecules (16–19). However, it is currently unknown whether HIV-*nef* may also promote immunodeficiency by blocking the phosphorylation of Y320 (44). If TLR signaling in APCs can indeed promote Y320 phosphorylation, it raises the intriguing possibility that subsets of intracellular MHC-I molecules trafficking through TLR-positive endocytic compartments bearing internalized TLR ligand-containing cargo could be simultaneously loaded with pathogen-derived peptides and “tagged” with pY320 to distinguish them from HLA-I complexes bearing self-peptides (37, 45–48). It will be interesting to explore whether such a tagging mechanism could contribute to the immune system’s ability to effectively discern self-antigens from non-self antigens displayed by the same APCs (49–51).

Although it is somewhat counter-intuitive that CD8+ T cells may “sense” and be influenced by cytoplasmic tail modifications to cognate HLA-I/peptide complexes, there are several potential mechanisms by which this could occur. For example, cytoplasmic tail modifications to MHC-I are well-known to influence the rate of internalization from the cell surface, which could impact the half-life of peptide presentation (2, 11, 20, 52–56). To test whether altered internalization might account for the priming differences observed with the Y320 mutation, we directly quantified SCT internalization in DC2.4 cells using both flow cytometry and confocal microscopy. Unexpectedly, neither approach revealed significant differences in internalization kinetics among the SCT variants. This contrasts with earlier reports that Y320 mutations affect internalization, which were conducted in other cell types. Because endocytosis of the same molecule can proceed through distinct mechanisms depending on the cellular context (57), our findings suggest that the impact of Y320 on internalization is cell-type dependent. Thus, at least in DC2.4 cells, the enhanced priming driven by Y320E cannot be explained by altered rates of SCT internalization.

Other mechanisms may account for the observed effects of Y320 modifications. Tail alterations could influence clustering or shedding of MHC-I/peptide complexes at the cell surface, with or without TCR engagement (10, 21, 26), or modulate interactions with the cytoskeleton and downstream signaling cascades (58, 59). Since antigen density, MHC-I cell surface mobility, and cytoskeletal interactions can all influence the strength of Signal 1 provided to CD8+ T cells upon TCR-based recognition, tail modifications could conceivably impact the way CTLs recognize and respond to cognate antigen on APCs (60, 61). For example, little is known regarding how tail modifications might influence cell surface localization, particularly their inclusion or exclusion within lipid rafts, or their ability to facilitate the co-localization of MHC-I/peptide complexes with co-stimulatory or co-inhibitory molecules known to influence T cell fate (4, 62–65). Lastly, since cognate CD8+ T cells have the capacity through trogocytosis to acquire specific MHC-I/peptide complexes that are subsequently observed on the T cell surface (66–68), the cytoplasmic domain and any associated APC modifications could be sensed from within, possibly influencing T cell fate long after their direct interaction with APCs (69). Further studies are required to determine the mechanism(s) by which MHC-I cytoplasmic tail modifications impact CD8+ T cell-mediated immunity (69).

In summary, our findings highlight a potentially significant role for Y320 phosphorylation in facilitating effective CTL priming and impacting T cell differentiation towards the promotion of memory responses. Our demonstration that Y320E-mutated MHC-I augments priming of both human and mouse CD8+ T cell responses underscores the translational potential of targeting this phosphorylation site as a nexus point through which to modulate CTL-based immune responses. Identifying the mammalian tyrosine kinases and phosphatases that act on the Y320 residue would be a promising first step towards this goal, since inhibition of one or the other could serve to inhibit or promote CTL responses, respectively. If pY320 indeed constitutes a “Go” signal for promoting CTL-mediated immunity, we speculate that its occurrence would likely be inhibited within cold tumor microenvironments but would potentially be augmented in lymph nodes during the initial stages of robust antiviral CTL responses. Future work should explore the potential of Y320E-based DC vaccines to enhance not only anti-tumor immunity but also the effectiveness of current viral vaccine platforms, possibly paving the way for inducing more robust and sustained CTL responses in diverse clinical settings.

## Materials and methods

### Mice and cell lines

C57BL/6J (CD45.1 and CD45.2) and OT-I mice were purchased from Jackson Laboratory, USA. The human macrophage cell line KG-1 (ATCC, Cat# CCL-246) was obtained from ATCC, USA. The mouse DC2.4 cell line (Sigma-Aldrich, Cat# SCC142) was purchased from Sigma-Aldrich, USA. The MC38 mouse tumor cell line was kindly provided by Dr. Yared Hailemichael at MD Anderson Cancer Center, USA. All cell lines were tested and confirmed to be mycoplasma negative.

### Single chain trimers and cell line transductions

cDNA sequences for HLA-A2 variants and for H-2Kb WT were synthesized by GenScript and inserted between NotI and ClaI sites into the pMG-eYFP retroviral vector. For the construction of single-chain trimers (SCT), sequences of HLA-A2 and H-2Kb were PCR-amplified from pMG-eYFP and inserted into pSBtet-Bla (Addgene plasmid #60510), with modification of their cytoplasmic tails so that WT, Y320E, and Y320F forms could be created by oligonucleotide insertion. InFusion cloning (Takara Bio) was then used to insert, between the MHC-I signal peptide and the mature MHC-I heavy chain, a gene block (Integrated DNA Technologies) containing elements of the SCT (25), from 5’ to 3’: a Type IIS enzyme cassette for “seamless” insertion of antigenic peptide by oligonucleotide cloning; a linker encoding GGGAS(G4S)_2_; cDNA for β2-microglobulin (β2mb); and a linker encoding (G4S)_4_. After insertion of the antigenic peptide (FluM1 [GILGFVFTL] or OVA [SIINFEKL]), the constructs transferred to a modified version of pHR_SFFV (Addgene plasmid #79121), downstream of cDNA for a puromycin resistance gene and a FMV 2A sequence. Lentivirus was prepared by transient transfection into 293T cells, using pCMV-VSV-G (Addgene plasmid #8454) and psPAX2 (Addgene plasmid #12260), and filtered supernatant was transduced by “spinoculation” with polybrene (8 mg/mL) at 1460xg for 90 min. SCT constructs will be deposited with Addgene.

### Human CD8+ T cell priming and expansion

Peripheral blood mononuclear cells (PBMCs) were isolated from buffy coats obtained from healthy blood donors at HemaCare. Written informed consent was obtained from all donors before inclusion. Isolation of PBMCs followed the protocol described by Eerkens et al. (70). PBMCs were plated at a density of 1.5 × 10_6_ cells/mL in 48-well plates. KG-1 cells expressing HLA-A2 with cytoplasmic tail mutations were pulsed with FluM1(58–66) peptide (30 μg/mL, GenScript, USA) or MART-1 peptide (30 μg/mL) for 1 hour, washed with PBS (Thermo Fisher Scientific, USA), and co-cultured with PBMCs. IL-21 (BioLegend, USA) was added every 3 days. On day 7, the PBMCs were re-plated, and KG-1 cells were pulsed with peptide (10 μg/mL) and reintroduced for a second stimulation. Two days later, IL-21 and IL-7 (BioLegend, USA) were added to the cultures. On day 14, cells were stained with anti-human CD8+ (BioLegend, USA) and FluM1 (MBL, Japan) or MART-1 tetramer and analyzed by flow cytometry.

### RNA sequencing and data analysis

CD8+ tetramer+ T cells after flow sorting were sent for RNA isolation and sequencing at Avera Genetics (Sioux Falls, SD). The quality checkup of raw reads was performed by FastQC (v0.11.5) and summarized by MultiQC (v1.7). FASTQ files were mapped to the human reference genome (GRCh38) using STAR (v2.7.10a) and RSEM (v1.3.3) with default parameter settings. Gene expression was quantified by TPM (Transcripts Per Kilobase Million) and transformed by log2(TPM + 1). Differential gene expression (DE) analysis was performed on R software (v4.3.2) using package DESeq2 (v1.40.2). GSEA was performed using R packages: msigdbr (v7.5.1) and fgsea (v1.28.0).

### Isolation of OT-I splenocytes and adoptive transfer

Spleens from OT-I mice were mechanically disrupted through a 70 μm strainer, and red blood cells were lysed using ACK Lysing Buffer (Gibco, USA). Mouse CD8+ T cells were isolated using the Naïve CD8+α+ T Cell Isolation Kit (StemCell Technologies, Canada, Cat# 19858) according to the manufacturer’s protocol. Isolated cells were resuspended at 1 × 10_5_ cells/mL in PBS and adoptively transferred into recipient mice (100 μL/mouse) via tail vein injection.

### DC2.4 vaccination and tumor inoculation

For tumor control experiments, mice were immunized twice at 7-day intervals with 1 × 10_6_ DC2.4 cells (irradiated at 2000 rads) expressing OVA single-chain trimer via subcutaneous injection into the right flank. One week after the final immunization, peripheral blood (50 μL) was collected via tail nicking and stained with H2Kb-OVA tetramer and anti-CD8 antibodies for flow cytometry. Subsequently, 2 × 10_6_ MC38-OVA cells were inoculated subcutaneously into the left flank. Tumor size was measured weekly with calipers and expressed as tumor area (mm²). Mice with tumor areas >150 mm² or bearing tumor ulceration were euthanized. Tumors and tumor-draining lymph nodes were collected on day 40 post-tumor inoculation for further analysis.

### Tumor infiltrating T cells, tumor draining lymph node, and splenocyte analysis

Single-cell suspensions were prepared from tumors, lymph nodes, or spleens by mechanical disruption followed by a 1-hour enzymatic digestion at 37 °C in RPMI-1640 medium (Thermo Fisher Scientific, USA) supplemented with 10% FCS, 100 U/mL DNase, 300 U/mL collagenase type I, and 60 U/mL hyaluronidase (Worthington Biochemical, USA). Digested tissues were filtered through nylon meshes (70 μm and 40 μm) and centrifuged. Cell suspensions were stained with antibodies from BD Pharmingen, USA; eBioscience, USA; or BioLegend, USA, and analyzed using an Aurora Blue cytometer (Cytek Biosciences, USA). Data were processed using FlowJo software (Tree Star, USA).

### Assays of MHC-I internalization

For flow cytometry, DC2.4 cells expressing different SCTs were initially labeled at 4 °C with APC-conjugated mouse antibody recognizing the H-2Kb–SIINFEKL combined epitope (BioLegend, Cat#141605). After washing, an aliquot of these cells underwent secondary labeling at 4 °C with an FITC-conjugated antibody recognizing primary antibody (BioLegend, Cat#406605). Separately, the remaining primarily-labeled cells were incubated at 37 °C for 60 min to allow spontaneous internalization, and then underwent secondary labeling, after which all cells were fixed and analyzed by flow cytometry. Internalization was quantified as the APC: FITC ratio of intensities for each cell.

Confocal microscopy was performed similarly, although the fluorophores were changed to overcome the problem of photobleaching. DC2.4 cells were sequentially labeled as described above. Primary labeling was done with Vio Bright B515-conjugated humanized antibody to H-2K^b–SIINFEKL (Miltenyi Biotec, Cat#130-116-919). Secondary labeling was done with Alexa Fluor 594-conjugated anti-human antibody (Jackson ImmunoResearch, Cat#AB_2337856), followed by mounting on slides using ProLong Glass Antifade Mountant with NucBlue counterstaining (Invitrogen, USA). Confocal imaging was performed on a Leica SP8 instrument.

## Data Availability

The raw data supporting the conclusions of this article will be made available by the authors, without undue reservation.

## Supplementary material

Supplementary Figure 1 Surface Expression of HLA-A*0201 constructs in KG-1 Cells. **(A)** Surface HLA-A*0201 expression was assessed using BB7.2-APC antibody staining. Expression of eYFP, encoded downstream of the same IRES element within the lentiviral vector, served as a surrogate for HLA-A*0201 transcript levels. MFI values for eYFP: Parental untransduced cells = 204, WT = 8,625, Y320F = 8,974, Y320E = 9,649. MFI values for APC: Parental = 113, WT = 17,589, Y320F = 12,238, Y320E = 13,530. **(B)** Schematic representation of the HLA-A*0201 single-chain trimer chimeric protein.

Supplementary Figure 2 Effect of HLA-A*0201 Y320 Mutations on Expression Level of Selected Genes in Human Primed Antigen-Specific CD8+ T Cells. PBMC from 3 healthy donors were stimulated twice with KG-1 cells expressing WT, Y320F, or Y320E SCTs bearing FluM1 peptide, then CD8+/tetramer+ cells were sorted for RNA-Seq. Each data point represents the value for an individual donor, with paired comparisons performed using a two-tailed paired Student’s t-test. Data are presented as mean ± standard error of the mean (SEM) from n = 3 donors.

Supplementary Figure 3 Surface OVA-Kb Expression and Cell Surface Internalization. **(A)** Schematics of the SCTs and surface expression of OVA-Kb on cells assessed by flow cytometry. **(B)** Flow cytometric analysis of surface H2-K^b–OVA SCT internalization. APC vs. FITC fluorescence ratios were measured at 0 and 60 min to quantify internalization across WT, Y320F, and Y320E constructs. Median APC/FITC ratios are shown in the accompanying tables. **(C)** Confocal microscopy images of surface H2-K^b–OVA SCT internalization at 0 and 60 min. Internalized molecules are labeled in green, surface-retained molecules are labeled in red (secondary antibody), and merged signals appear orange. Nuclei are counterstained in blue.

Supplementary Figure 4 Phenotypic Analysis of OVA-Specific T Cells Post-Vaccination. **(A)** Relative frequency of CD127+ and **(B)** KLRG1+ cells in donor and endogenous peripheral OVA specific T cells across different vaccination groups at various time points. Statistical differences between Y320 mutants were assessed using one-way ANOVA, followed by pairwise comparisons using an unpaired two-tailed t-test; only significant p values (p < 0.05) are shown. Data are presented as mean ± standard error of the mean (SEM).

Supplementary Figure 5 Splenic T cell phenotypes following DC2.4/SCT vaccination. **(A)** Schematic representation of the vaccination protocol. Mice were injected subcutaneously with 1×10^6 irradiated (2000 rads) DC2.4 cells on day 1, and spleens were collected on day 7. **(B)** Frequencies of OVA-specific CD8+ T cells and distribution of distinct OVA-specific CD8+ T cell subsets in spleens from mice vaccinated with parental, WT, Y320F, or Y320E DC2.4-OVA-SCT constructs. Subsets shown include Tem (CD62L–, KLRG1-, CD127lo), Teff (CD62L–, KLRG1+, CD127lo), and Tcm (CD62L+, KLRG1–, CD127hi) populations. Data are presented as mean ± SEM. Statistical differences between Y320 variant groups were assessed using one-way ANOVA.

Supplementary Figure 6 Surface Marker Expression of Tumor-infiltrating CD8+ T Cells in DC2.4/SCT-vaccinated Mice. Statistical differences between Y320 groups were assessed using one-way ANOVA, followed by pairwise comparisons using an unpaired two-tailed t-test; only significant p values (p < 0.05) are shown. Data are presented as mean ± standard error of the mean (SEM).

## References

[1] Lizee G Basha G Tiong J Julien JP Tian M Biron KE. Control of dendritic cell cross-presentation by the major histocompatibility complex class I cytoplasmic domain. Nat Immunol. (2003) 4:1065–73. doi: 10.1038/ni989, PMID: 14566337

[2] Lizee G Basha G Jefferies WA. Tails of wonder: endocytic-sorting motifs key for exogenous antigen presentation. Trends Immunol. (2005) 26:141–9. doi: 10.1016/j.it.2005.01.005, PMID: 15745856

[3] Hornbeck PV Zhang B Murray B Kornhauser JM Latham V Skrzypek E. PhosphoSitePlus, 2014: mutations, PTMs and recalibrations. Nucleic Acids Res. (2015) 43:D512–20. doi: 10.1093/nar/gku1267, PMID: 25514926 PMC4383998

[4] Santos SG Powis SJ Arosa FA. Misfolding of major histocompatibility complex class I molecules in activated T cells allows cis-interactions with receptors and signaling molecules and is associated with tyrosine phosphorylation. J Biol Chem. (2004) 279:53062–70. doi: 10.1074/jbc.M408794200, PMID: 15471856

[5] Guild BC Strominger JL. Human and murine class I MHC antigens share conserved serine 335, the site of HLA phosphorylation *in vivo* . J Biol Chem. (1984) 259:9235–40. doi: 10.1016/S0021-9258(17)47290-5, PMID: 6430898

[6] Peyron JF Fehlmann M. Phosphorylation of class I histocompatibility antigens in human B lymphocytes. Regulation by phorbol esters and insulin. Biochem J. (1988) 256:p. doi: 10.1042/bj2560763, PMID: 3066355 PMC1135481

[7] Guild BC Erikson RL Strominger JL. HLA-A2 and HLA-B7 antigens are phosphorylated *in vitro* by rous sarcoma virus kinase (pp60v-src) at a tyrosine residue encoded in a highly conserved exon of the intracellular domain. Proc Natl Acad Sci U.S.A. (1983) 80:2894–8. doi: 10.1073/pnas.80.10.2894, PMID: 6304688 PMC393939

[8] Pober JS Guild BC Strominger JL. Phosphorylation *in vivo* and *in vitro* of human histocompatibility antigens (HLA-A and HLA-B) in the carboxy-terminal intracellular domain. Proc Natl Acad Sci U.S.A. (1978) 75:6002–6. doi: 10.1073/pnas.75.12.6002, PMID: 282620 PMC393105

[9] Capps GG Zuniga MC. Phosphorylation of class I MHC molecules in the absence of phorbol esters is an intracellular event and may be characteristic of trafficking molecules. Mol Immunol. (2000) 37:59–71. doi: 10.1016/S0161-5890(00)00019-5, PMID: 10781836

[10] Rodriguez-Cruz TG Liu S Khalili JS Whittington M Zhang M Overwijk W. Natural splice variant of MHC class I cytoplasmic tail enhances dendritic cell-induced CD8+ T-cell responses and boosts anti-tumor immunity. PloS One. (2011) 6:e22939. doi: 10.1371/journal.pone.0022939, PMID: 21860662 PMC3157908

[11] Bradley SD Chen Z Melendez B Talukder A Khalili JS Rodriguez-Cruz T. BRAFV600E co-opts a conserved MHC class I internalization pathway to diminish antigen presentation and CD8+ T-cell recognition of melanoma. Cancer Immunol Res. (2015) 3:602–9. doi: 10.1158/2326-6066.CIR-15-0030, PMID: 25795007 PMC4457616

[12] Bian Y Song C Cheng K Dong M Wang F Huang J. An enzyme assisted RP-RPLC approach for in-depth analysis of human liver phosphoproteome. J Proteomics. (2014) 96:253–62. doi: 10.1016/j.jprot.2013.11.014, PMID: 24275569

[13] Iliuk AB Martin VA Alicie BM Geahlen RL Tao WA. In-depth analyses of kinase-dependent tyrosine phosphoproteomes based on metal ion-functionalized soluble nanopolymers. Mol Cell Proteomics. (2010) 9:2162–72. doi: 10.1074/mcp.M110.000091, PMID: 20562096 PMC2953913

[14] Schweppe DK Rigas JR Gerber SA. Quantitative phosphoproteomic profiling of human non-small cell lung cancer tumors. J Proteomics. (2013) 91:286–96. doi: 10.1016/j.jprot.2013.07.023, PMID: 23911959 PMC3825743

[15] Xu S Liu X Bao Y Zhu X Han C Zhang P. Constitutive MHC class I molecules negatively regulate TLR-triggered inflammatory responses via the Fps-SHP-2 pathway. Nat Immunol. (2012) 13:551–9. doi: 10.1038/ni.2283, PMID: 22522491

[16] Williams MA Trout R Spector SA. HIV-1 gp120 modulates the immunological function and expression of accessory and co-stimulatory molecules of monocyte-derived dendritic cells. J Hematother Stem Cell Res. (2002) 11:829–47. doi: 10.1089/152581602760404630, PMID: 12427289

[17] Roeth JF Williams M Kasper MR Filzen TM Collins KL Nef disrupts trafficking by recruiting to the cytoplasmic tail HIV-1MHC-IAP-1MHC-I. HIV-1 Nef disrupts MHC-I trafficking by recruiting AP-1 to the MHC-I cytoplasmic tail. J Cell Biol. (2004) 167:903–13. doi: 10.1083/jcb.200407031, PMID: 15569716 PMC2172469

[18] Le Gall M Grall D Chambard JC Pouyssegur J Van Obberghen-Schilling E An anchorage-dependent signal distinct from kinase activation is required for cell cycle progression MAP. An anchorage-dependent signal distinct from p42/44 MAP kinase activation is required for cell cycle progression. Oncogene. (1998) 17:1271–7. doi: 10.1038/sj.onc.1202057, PMID: 9771970

[19] Le Gall S Buseyne F Trocha A Walker BD Heard JM Schwartz O. Distinct trafficking pathways mediate Nef-induced and clathrin-dependent major histocompatibility complex class I down-regulation. J Virol. (2000) 74:9256–66. doi: 10.1128/JVI.74.19.9256-9266.2000, PMID: 10982373 PMC102125

[20] Basha G Lizee G Reinicke AT Seipp RP Omilusik KD Jefferies WA. MHC class I endosomal and lysosomal trafficking coincides with exogenous antigen loading in dendritic cells. PloS One. (2008) 3:e3247. doi: 10.1371/journal.pone.0003247, PMID: 18802471 PMC2532750

[21] Santos SG Antoniou AN Sampaio P Powis SJ Arosa FA Lack of tyrosine impairs spontaneous endocytosis and enhances release of molecules HLA-B27. Lack of tyrosine 320 impairs spontaneous endocytosis and enhances release of HLA-B27 molecules. J Immunol. (2006) 176:2942–9. doi: 10.4049/jimmunol.176.5.2942, PMID: 16493052

[22] Bonifacino JS Traub LM. Signals for sorting of transmembrane proteins to endosomes and lysosomes. Annu Rev Biochem. (2003) 72:395–447. doi: 10.1146/annurev.biochem.72.121801.161800, PMID: 12651740

[23] Stateva SR Salas V Benaim G Menendez M Solis D Villalobo A. Characterization of phospho-(tyrosine)-mimetic calmodulin mutants. PloS One. (2015) 10:e0120798. doi: 10.1371/journal.pone.0120798, PMID: 25830911 PMC4382182

[24] Umeshappa CS Huang H Xie Y Wei Y Mulligan SJ Deng Y. CD4+ Th-APC with acquired peptide/MHC class I and II complexes stimulate type 1 helper CD4+ and central memory CD8+ T cell responses. J Immunol. (2009) 182:193–206. doi: 10.4049/jimmunol.182.1.193, PMID: 19109150

[25] Yu YY Netuschil N Lybarger L Connolly JM Hansen TH Cutting edge: single-chain trimers of class molecules form stable structures that potently stimulate antigen-specific cells and cells MHCITB. Cutting edge: single-chain trimers of MHC class I molecules form stable structures that potently stimulate antigen-specific T cells and B cells. J Immunol. (2002) 168:3145–9. doi: 10.4049/jimmunol.168.7.3145, PMID: 11907065

[26] Brockmeyer C Paster W Pepper D Tan CP Trudgian DC McGowan S. T cell receptor (TCR)-induced tyrosine phosphorylation dynamics identifies THEMIS as a new TCR signalosome component. J Biol Chem. (2011) 286:7535–47. doi: 10.1074/jbc.M110.201236, PMID: 21189249 PMC3045008

[27] Pittet MJ Valmori D Dunbar PR Speiser DE Lienard D Lejeune F. High frequencies of naive Melan-A/MART-1-specific CD8(+) T cells in a large proportion of human histocompatibility leukocyte antigen (HLA)-A2 individuals. J Exp Med. (1999) 190:705–15. doi: 10.1084/jem.190.5.705, PMID: 10477554 PMC2195613

[28] Choudhuri K Wiseman D Brown MH Gould K van der Merwe PA receptor triggering is critically dependent on the dimensions of its peptide-MHC ligand T-cell. T-cell receptor triggering is critically dependent on the dimensions of its peptide-MHC ligand. Nature. (2005) 436:578–82. doi: 10.1038/nature03843, PMID: 16049493

[29] Kim S Poursine-Laurent J Truscott SM Lybarger L Song YJ Yang L. Licensing of natural killer cells by host major histocompatibility complex class I molecules. Nature. (2005) 436:709–13. doi: 10.1038/nature03847, PMID: 16079848

[30] Wang B Primeau TM Myers N Rohrs HW Gross ML Lybarger L. A single peptide-MHC complex positively selects a diverse and specific CD8 T cell repertoire. Science. (2009) 326:871–4. doi: 10.1126/science.1177627, PMID: 19892989 PMC2828816

[31] Almeida L Lochner M Berod L Sparwasser T Metabolic pathways in cell activation and lineage differentiation T. Metabolic pathways in T cell activation and lineage differentiation. Semin Immunol. (2016) 28:514–24. doi: 10.1016/j.smim.2016.10.009, PMID: 27825556

[32] Naito Y Koyama S Masuhiro K Hirai T Uenami T Inoue T. Tumor-derived semaphorin 4A improves PD-1-blocking antibody efficacy by enhancing CD8(+) T cell cytotoxicity and proliferation. Sci Adv. (2023) 9:eade0718. doi: 10.1126/sciadv.ade0718, PMID: 37205755 PMC10198637

[33] Shrestha S Yang K Wei J Karmaus PW Neale G Chi H. Tsc1 promotes the differentiation of memory CD8+ T cells via orchestrating the transcriptional and metabolic programs. Proc Natl Acad Sci U.S.A. (2014) 111:14858–63. doi: 10.1073/pnas.1404264111, PMID: 25271321 PMC4205612

[34] Tang Y Chen Z Zuo Q Kang Y Regulation of cells by lipid metabolism in cancer progression CD8+T. Regulation of CD8+ T cells by lipid metabolism in cancer progression. Cell Mol Immunol. (2024) 21:1215–30. doi: 10.1038/s41423-024-01224-z, PMID: 39402302 PMC11527989

[35] van der Windt GJ Pearce EL. Metabolic switching and fuel choice during T-cell differentiation and memory development. Immunol Rev. (2012) 249:27–42. doi: 10.1111/j.1600-065X.2012.01150.x, PMID: 22889213 PMC3645891

[36] Hargadon KM. Murine and human model systems for the study of dendritic cell immunobiology. Int Rev Immunol. (2016) 35:85–115. doi: 10.3109/08830185.2014.952413, PMID: 25203775

[37] Kovacsovics-Bankowski M Rock KL. A phagosome-to-cytosol pathway for exogenous antigens presented on MHC class I molecules. Science. (1995) 267:243–6. doi: 10.1126/science.7809629, PMID: 7809629

[38] Okada N Tsujino M Hagiwara Y Tada A Tamura Y Mori K. Administration route-dependent vaccine efficiency of murine dendritic cells pulsed with antigens. Br J Cancer. (2001) 84:1564–70. doi: 10.1054/bjoc.2001.1801, PMID: 11384109 PMC2363668

[39] Young Chung J Thone MN Davies JE Gach JS Huw Davies D Forthal DN. Vaccination against SARS-CoV-2 using extracellular blebs derived from sp*ike protein-expressing dendritic cells* . Cell Immunol. (2023) 386:104691. doi: 10.1016/j.cellimm.2023.104691, PMID: 36822152 PMC9933546

[40] Pinchuk LM Filipov NM. Differential effects of age on circulating and splenic leukocyte populations in C57BL/6 and BALB/c male mice. Immun Ageing. (2008) 5:1. doi: 10.1186/1742-4933-5-1, PMID: 18267021 PMC2268915

[41] Baaten BJG Cooper AM Swain SL Bradley LM. Location, location, location: the impact of migratory heterogeneity on T cell function. Front Immunol. (2013) 4:311. doi: 10.3389/fimmu.2013.00311, PMID: 24115949 PMC3792444

[42] Blattman JN Antia R Sourdive DJ Wang X Kaech SM Murali-Krishna K. Estimating the precursor frequency of naive antigen-specific CD8 T cells. J Exp Med. (2002) 195:657–64. doi: 10.1084/jem.20001021, PMID: 11877489 PMC2193761

[43] Nesbeth Y Conejo-Garcia JR. Harnessing the effect of adoptively transferred tumor-reactive T cells on endogenous (host-derived) antitumor immunity. Clin Dev Immunol 2010. (2010) p:139304. doi: 10.1155/2010/139304, PMID: 21076522 PMC2975067

[44] Kulpa DA Del Cid N Peterson KA Collins KL Adaptor protein promotes cross-presentation through the same tyrosine signal in major histocompatibility complex class as that targeted by IHIV-1. Adaptor protein 1 promotes cross-presentation through the same tyrosine signal in major histocompatibility complex class I as that targeted by HIV-1. J Virol. (2013) 87:8085–98. doi: 10.1128/JVI.00701-13, PMID: 23678182 PMC3700188

[45] Houde M Bertholet S Gagnon E Brunet S Goyette G Laplante A. Phagosomes are competent organelles for antigen cross-presentation. Nature. (2003) 425:402–6. doi: 10.1038/nature01912, PMID: 14508490

[46] Guermonprez P Saveanu L Kleijmeer M Davoust J Van Endert P Amigorena S. ER-phagosome fusion defines an MHC class I cross-presentation compartment in dendritic cells. Nature. (2003) 425:397–402. doi: 10.1038/nature01911, PMID: 14508489

[47] Palliser D Guillen E Ju M Eisen HN. Multiple intracellular routes in the cross-presentation of a soluble protein by murine dendritic cells. J Immunol. (2005) 174:1879–87. doi: 10.4049/jimmunol.174.4.1879, PMID: 15699114

[48] Gromme M Uytdehaag FG Janssen H Calafat J van Binnendijk RS Kenter MJ. Recycling MHC class I molecules and endosomal peptide loading. Proc Natl Acad Sci U.S.A. (1999) 96:10326–31. doi: 10.1073/pnas.96.18.10326, PMID: 10468607 PMC17887

[49] Burgdorf S Scholz C Kautz A Tampe R Kurts C. Spatial and mechanistic separation of cross-presentation and endogenous antigen presentation. Nat Immunol. (2008) 9:558–66. doi: 10.1038/ni.1601, PMID: 18376402

[50] Gonzalez S Gonzalez-Rodriguez AP Suarez-Alvarez B Lopez-Soto A Huergo-Zapico L Lopez-Larrea C. Conceptual aspects of self and nonself discrimination. Self Nonself. (2011) 2:19–25. doi: 10.4161/self.2.1.15094, PMID: 21776331 PMC3136900

[51] Apcher S Vojtesek B Fahraeus R. In search of the cell biology for self- versus non-self- recognition. Curr Opin Immunol. (2023) 83:102334. doi: 10.1016/j.coi.2023.102334, PMID: 37210933

[52] Vega MA Strominger JL. Constitutive endocytosis of HLA class I antigens requires a specific portion of the intracytoplasmic tail that shares structural features with other endocytosed molecules. Proc Natl Acad Sci U.S.A. (1989) 86:2688–92. doi: 10.1073/pnas.86.8.2688, PMID: 2495533 PMC286983

[53] Tse DB Pernis B. Spontaneous internalization of Class I major histocompatibility complex molecules in T lymphoid cells. J Exp Med. (1984) 159:193–207. doi: 10.1084/jem.159.1.193, PMID: 6363594 PMC2187213

[54] Machy P Truneh A Gennaro D Hoffstein S Major histocompatibility complex class molecules internalized via coated pits in lymphocytes IT. Major histocompatibility complex class I molecules internalized via coated pits in T lymphocytes. Nature. (1987) 328:724–6. doi: 10.1038/328724a0, PMID: 2886920

[55] Dasgupta JD Watkins S Slayter H Yunis EJ Receptor-like nature of class endocytosis via coated pits IHLA:. Receptor-like nature of class I HLA: endocytosis via coated pits. J Immunol. (1988) 141:2577–80. doi: 10.4049/jimmunol.141.8.2577, PMID: 2902138

[56] Capps GG Van Kampen M Ward CL Zuniga MC Endocytosis of the class major histocompatibility antigen via phorbol myristate acetate-inducible pathway is cell-specific phenomenon and requires the cytoplasmic domain I. Endocytosis of the class I major histocompatibility antigen via a phorbol myristate acetate-inducible pathway is a cell-specific phenomenon and requires the cytoplasmic domain. J Cell Biol. (1989) 108:1317–29. doi: 10.1083/jcb.108.4.1317, PMID: 2925787 PMC2115514

[57] Rennick JJ Johnston APR Parton RG. Key principles and methods for studying the endocytosis of biological and nanoparticle therapeutics. Nat Nanotechnol. (2021) 16:266–76. doi: 10.1038/s41565-021-00858-8, PMID: 33712737

[58] Gur H Geppert TD Lipsky PE. Structural analysis of class I MHC molecules: the cytoplasmic domain is not required for cytoskeletal association, aggregation and internalization. Mol Immunol. (1997) 34:125–32. doi: 10.1016/S0161-5890(97)00007-2, PMID: 9188845

[59] Geppert TD Davis LS Gur H Wacholtz MC Lipsky PE Accessory cell signals involved in activation T-cell. Accessory cell signals involved in T-cell activation. Immunol Rev. (1990) 117:5–66. doi: 10.1111/j.1600-065X.1990.tb00566.x, PMID: 2147918

[60] Barbour S Edidin M. Cell-specific constraints to the lateral diffusion of a membrane glycoprotein. J Cell Physiol. (1992) 150:526–33. doi: 10.1002/jcp.1041500313, PMID: 1537882

[61] Vigano S Utzschneider DT Perreau M Pantaleo G Zehn D Harari A. Functional avidity: a measure to predict the efficacy of effector T cells? Clin Dev Immunol. (2012) 2012:153863. doi: 10.1155/2012/153863, PMID: 23227083 PMC3511839

[62] Mocsar G Volko J Ronnlund D Widengren J Nagy P Szollosi J. MHC I expression regulates co-clustering and mobility of interleukin-2 and -15 receptors in T cells. Biophys J. (2016) 111:100–12. doi: 10.1016/j.bpj.2016.05.044, PMID: 27410738 PMC4944762

[63] Anderson HA Roche PA. MHC class II association with lipid rafts on the antigen presenting cell surface. Biochim Biophys Acta. (2015) 1853:775–80. doi: 10.1016/j.bbamcr.2014.09.019, PMID: 25261705 PMC4344880

[64] Franzese O. Tumor microenvironment drives the cross-talk between co-stimulatory and inhibitory molecules in tumor-infiltrating lymphocytes: implications for optimizing immunotherapy outcomes. Int J Mol Sci. (2024) 25(23). doi: 10.3390/ijms252312848, PMID: 39684559 PMC11641238

[65] Chiu I Davis DM Strominger JL. Trafficking of spontaneously endocytosed MHC proteins. Proc Natl Acad Sci U.S.A. (1999) 96:13944–9. doi: 10.1073/pnas.96.24.13944, PMID: 10570178 PMC24170

[66] Huang JF Yang Y Sepulveda H Shi W Hwang I Peterson PA. TCR-Mediated internalization of peptide-MHC complexes acquired by T cells. Science. (1999) 286:952–4. doi: 10.1126/science.286.5441.952, PMID: 10542149

[67] Martinez-Martin N Alarcon B. Physiological and therapeutic relevance of T cell receptor-mediated antigen trogocytosis. BioMed J. (2024) 47:100630. doi: 10.1016/j.bj.2023.100630, PMID: 37459965 PMC11401223

[68] Uzana R Eisenberg G Sagi Y Frankenburg S Merims S Amariglio N. Trogocytosis is a gateway to characterize functional diversity in melanoma-specific CD8+ T cell clones. J Immunol. (2012) 188:632–40. doi: 10.4049/jimmunol.1101429, PMID: 22156347

[69] Reed J Wetzel SA. Trogocytosis-mediated intracellular signaling in CD4(+) T cells drives T(H)2-associated effector cytokine production and differentiation. J Immunol. (2019) 202:2873–87. doi: 10.4049/jimmunol.1801577, PMID: 30962293 PMC6504583

[70] Eerkens AL Vledder A van Rooij N Foijer F Nijman HW de Bruyn M. Rapid and efficient generation of antigen-specific isogenic T cells from cryopreserved blood samples. Immunol Cell Biol. (2022) 100:285–95. doi: 10.1111/imcb.12538, PMID: 35194830 PMC9314923

