# Supplementary material for: Mutation of conserved MHC class I cytoplasmic tyrosine affects CD8+ T cell priming, effector function, and memory response
Source: Front Immunol. 2025 Sep 30;16:1572342. doi: 10.3389/fimmu.2025.1572342 (PMC12518231; doi:10.3389/fimmu.2025.1572342)
Supplement: Supplementary Figure 1 — Surface Expression of HLA-A*0201 constructs in KG-1 Cells. (A) Surface HLA-A*0201 expression was assessed using BB7.2-APC antibody staining. Expression of eYFP, encoded downstream of the same IRES element within the lentiviral vector, served as a surrogate for HLA-A*0201 transcript levels. MFI values for eYFP: Parental untransduced cells = 204, WT = 8,625, Y320F = 8,974, Y320E = 9,649. MFI values for APC: Parental = 113, WT = 17,589, Y320F = 12,238, Y320E = 13,530. (B) Schematic representation of the HLA-A*0201 single-chain trimer chimeric protein. [file Presentation1.pptx]

## Slide 1
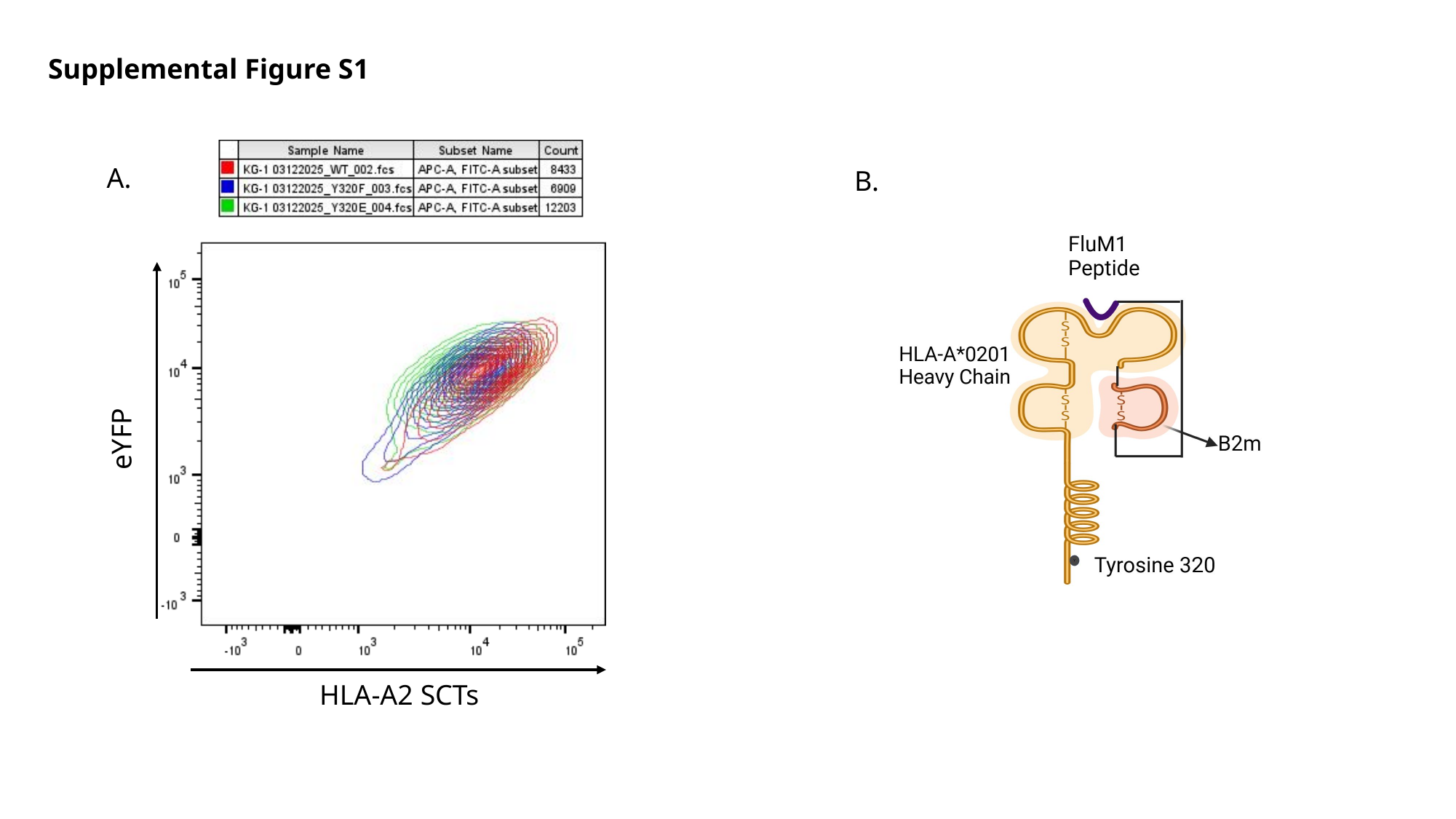

Supplemental Figure S1
B.
eYFP
HLA-A2 SCTs
A.

## Slide 2
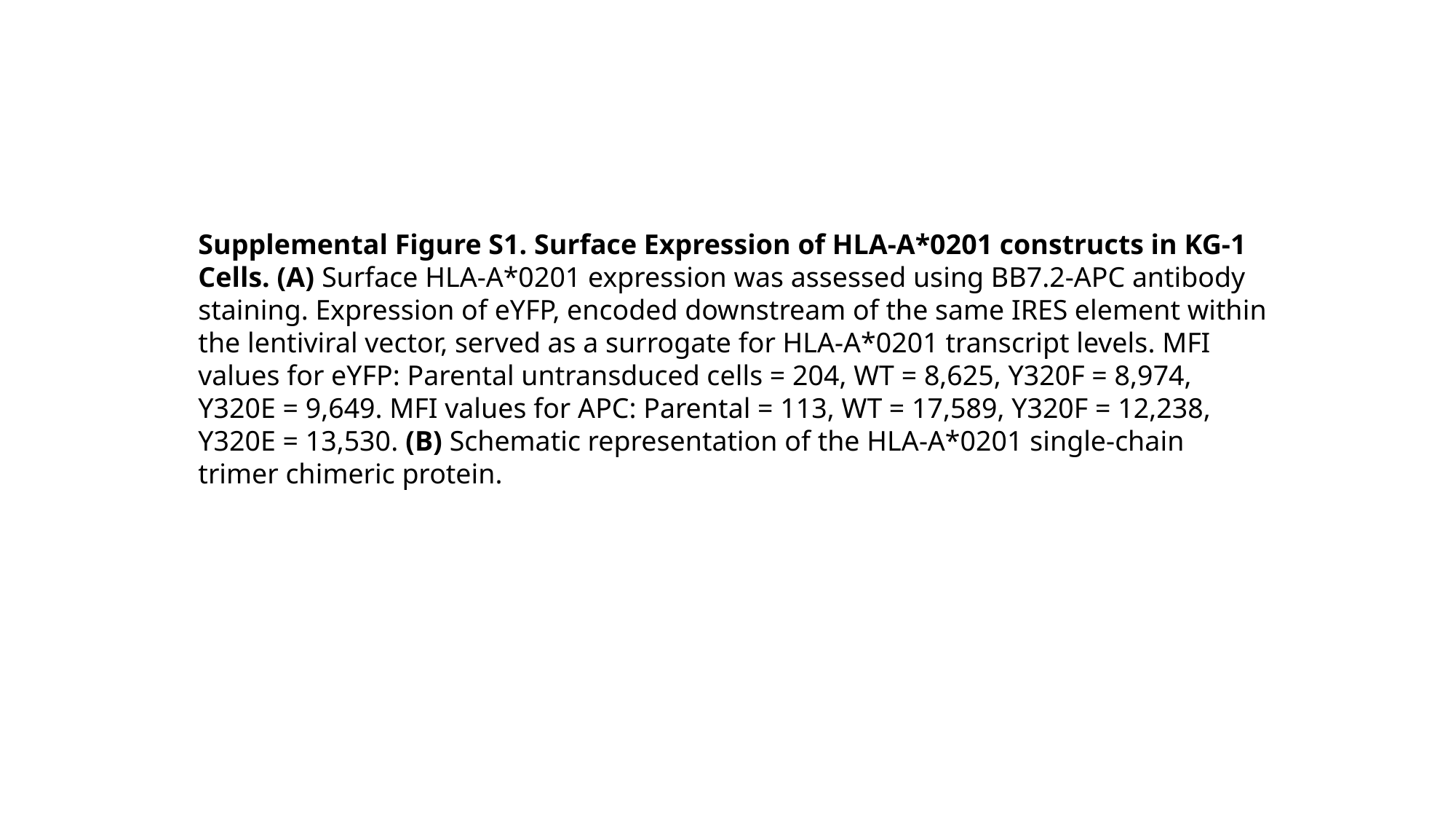

Supplemental Figure S1. Surface Expression of HLA-A*0201 constructs in KG-1 Cells. (A) Surface HLA-A*0201 expression was assessed using BB7.2-APC antibody staining. Expression of eYFP, encoded downstream of the same IRES element within the lentiviral vector, served as a surrogate for HLA-A*0201 transcript levels. MFI values for eYFP: Parental untransduced cells = 204, WT = 8,625, Y320F = 8,974, Y320E = 9,649. MFI values for APC: Parental = 113, WT = 17,589, Y320F = 12,238, Y320E = 13,530. (B) Schematic representation of the HLA-A*0201 single-chain trimer chimeric protein.

## Slide 3
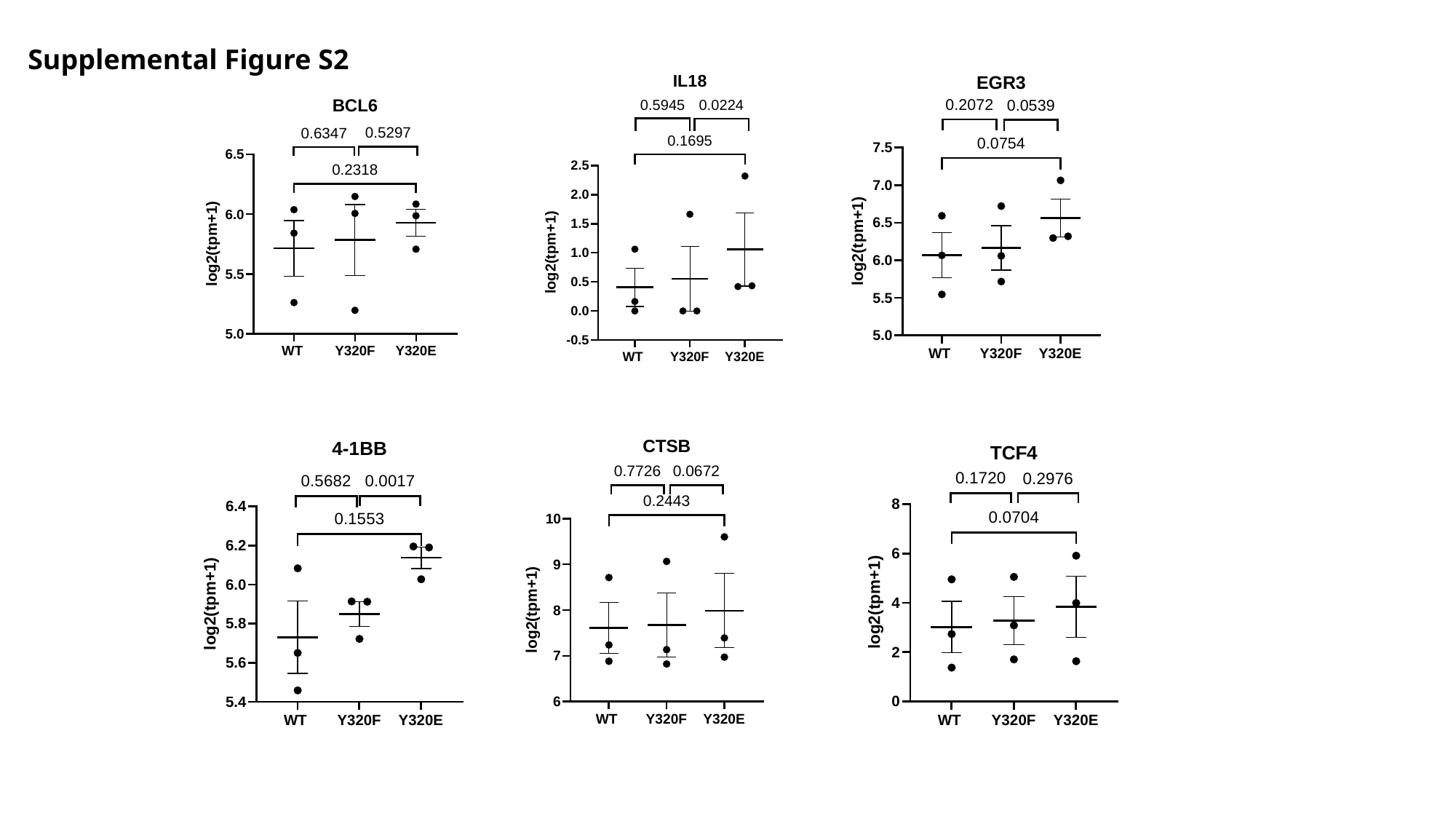

Supplemental Figure S2

## Slide 4
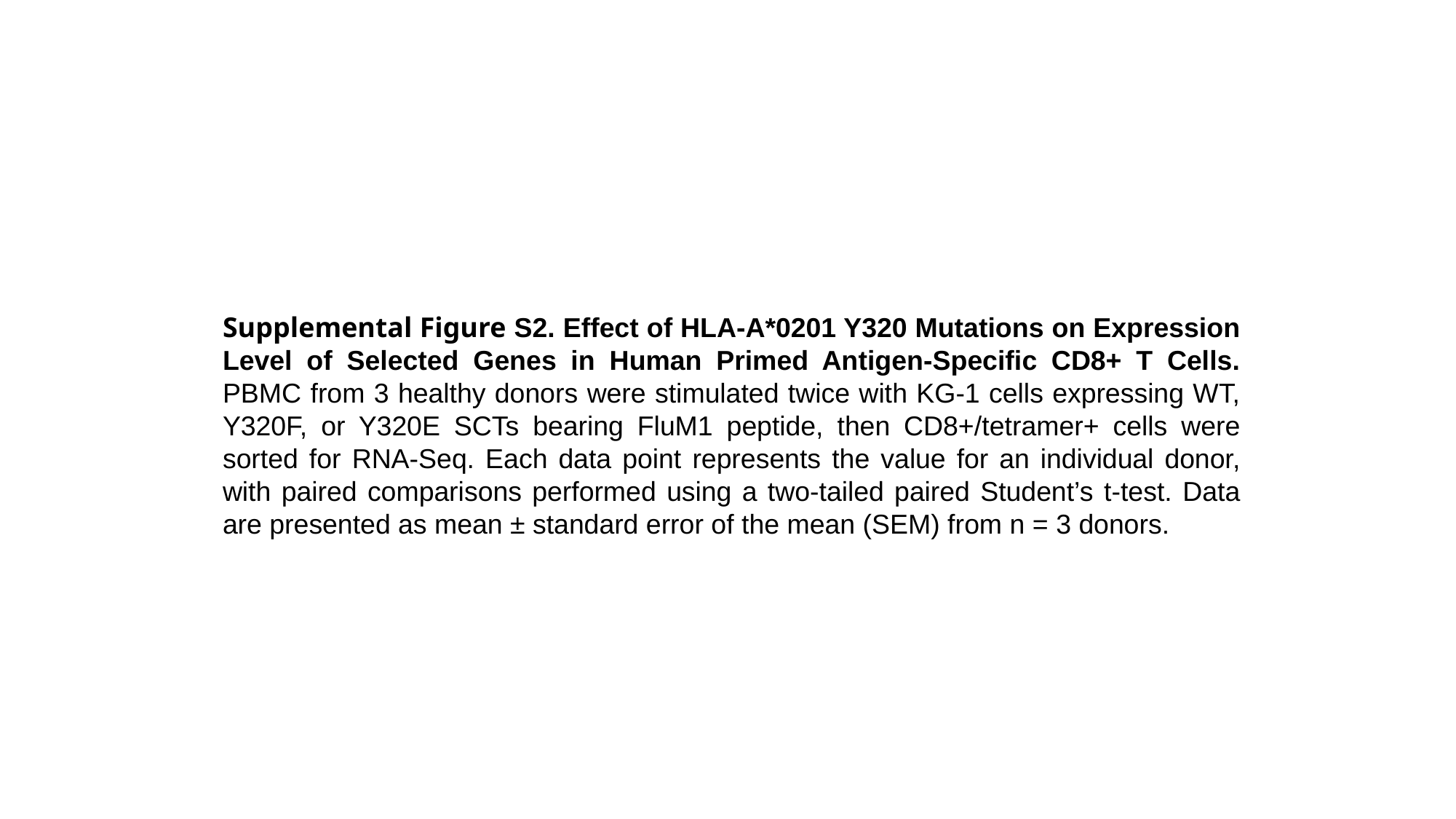

Supplemental Figure S2. Effect of HLA-A*0201 Y320 Mutations on Expression Level of Selected Genes in Human Primed Antigen-Specific CD8+ T Cells. PBMC from 3 healthy donors were stimulated twice with KG-1 cells expressing WT, Y320F, or Y320E SCTs bearing FluM1 peptide, then CD8+/tetramer+ cells were sorted for RNA-Seq. Each data point represents the value for an individual donor, with paired comparisons performed using a two-tailed paired Student’s t-test. Data are presented as mean ± standard error of the mean (SEM) from n = 3 donors.

## Slide 5
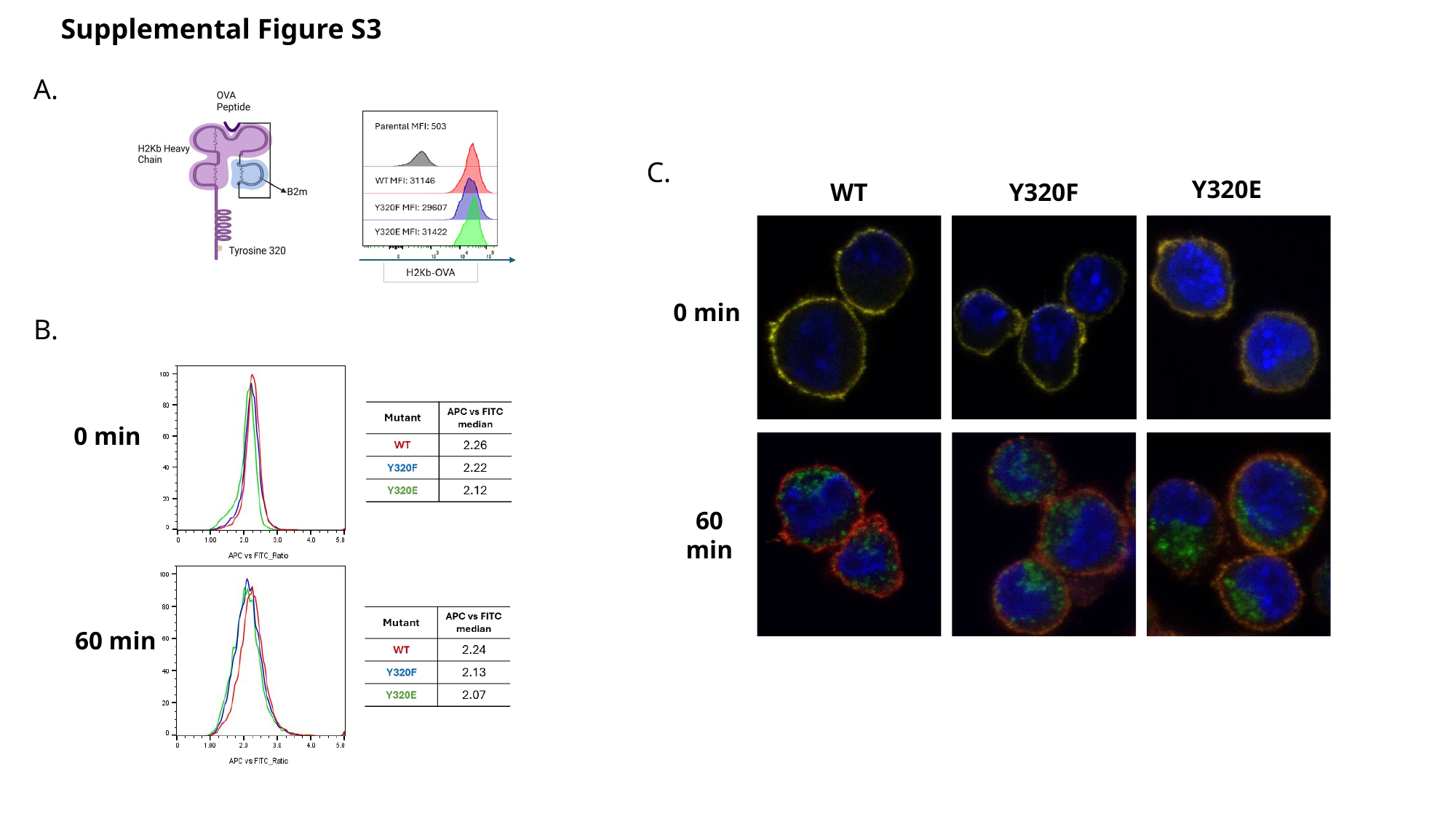

Supplemental Figure S3
A.
C.
Y320E
WT
Y320F
0 min
B.
0 min
60 min
60 min

## Slide 6
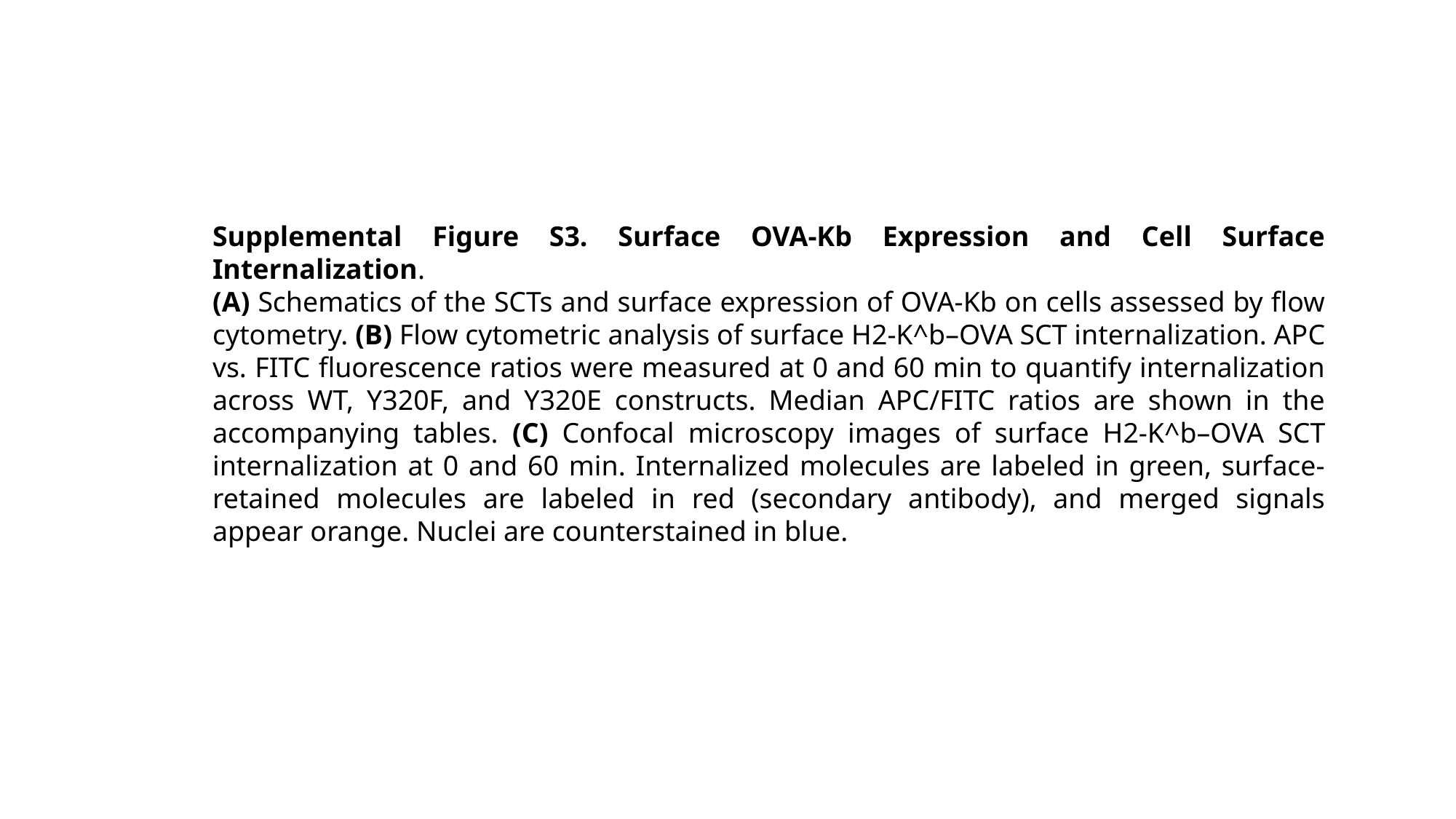

Supplemental Figure S3. Surface OVA-Kb Expression and Cell Surface Internalization.
(A) Schematics of the SCTs and surface expression of OVA-Kb on cells assessed by flow cytometry. (B) Flow cytometric analysis of surface H2-K^b–OVA SCT internalization. APC vs. FITC fluorescence ratios were measured at 0 and 60 min to quantify internalization across WT, Y320F, and Y320E constructs. Median APC/FITC ratios are shown in the accompanying tables. (C) Confocal microscopy images of surface H2-K^b–OVA SCT internalization at 0 and 60 min. Internalized molecules are labeled in green, surface-retained molecules are labeled in red (secondary antibody), and merged signals appear orange. Nuclei are counterstained in blue.

## Slide 7
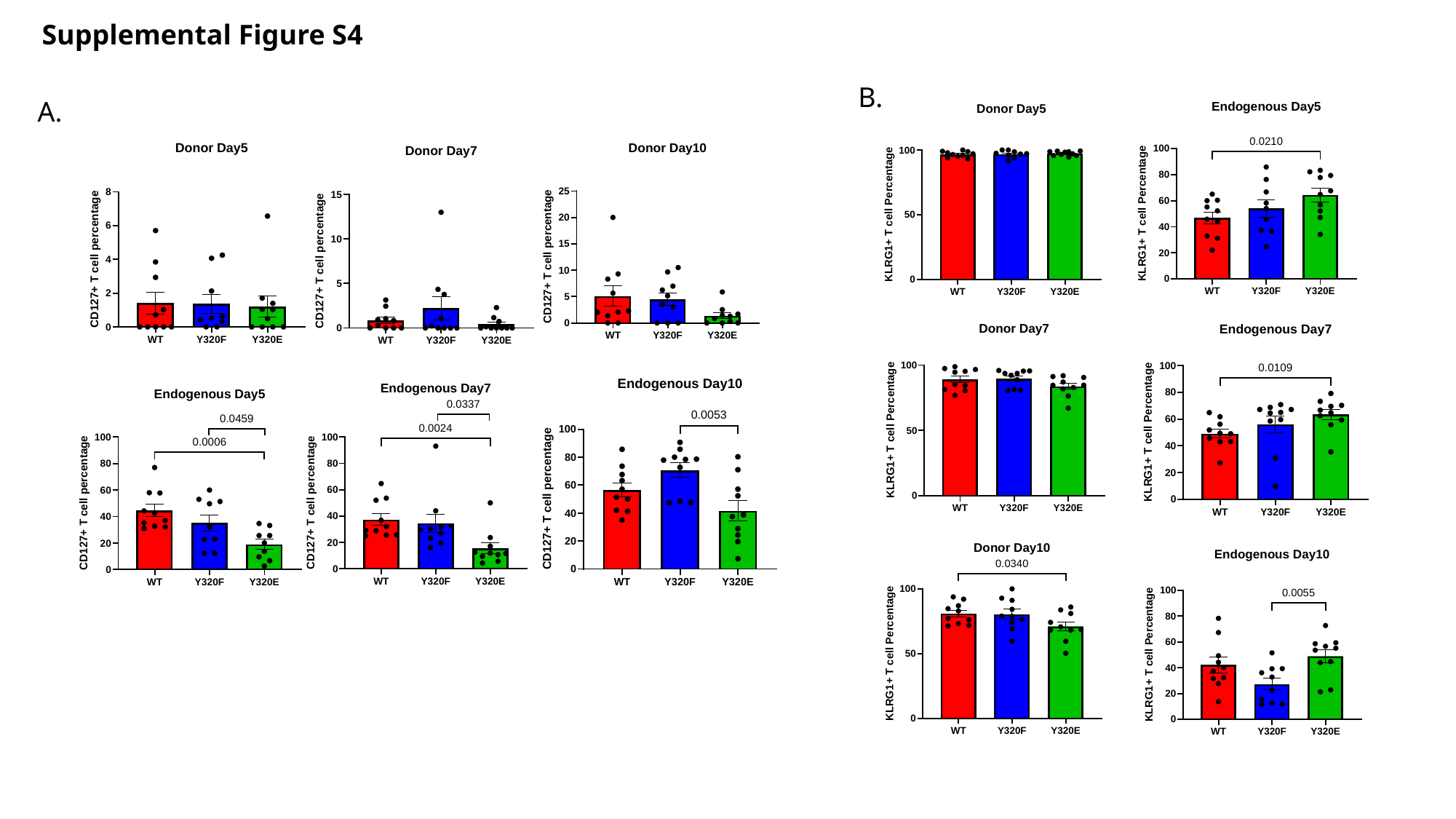

Supplemental Figure S4
B.
A.

## Slide 8
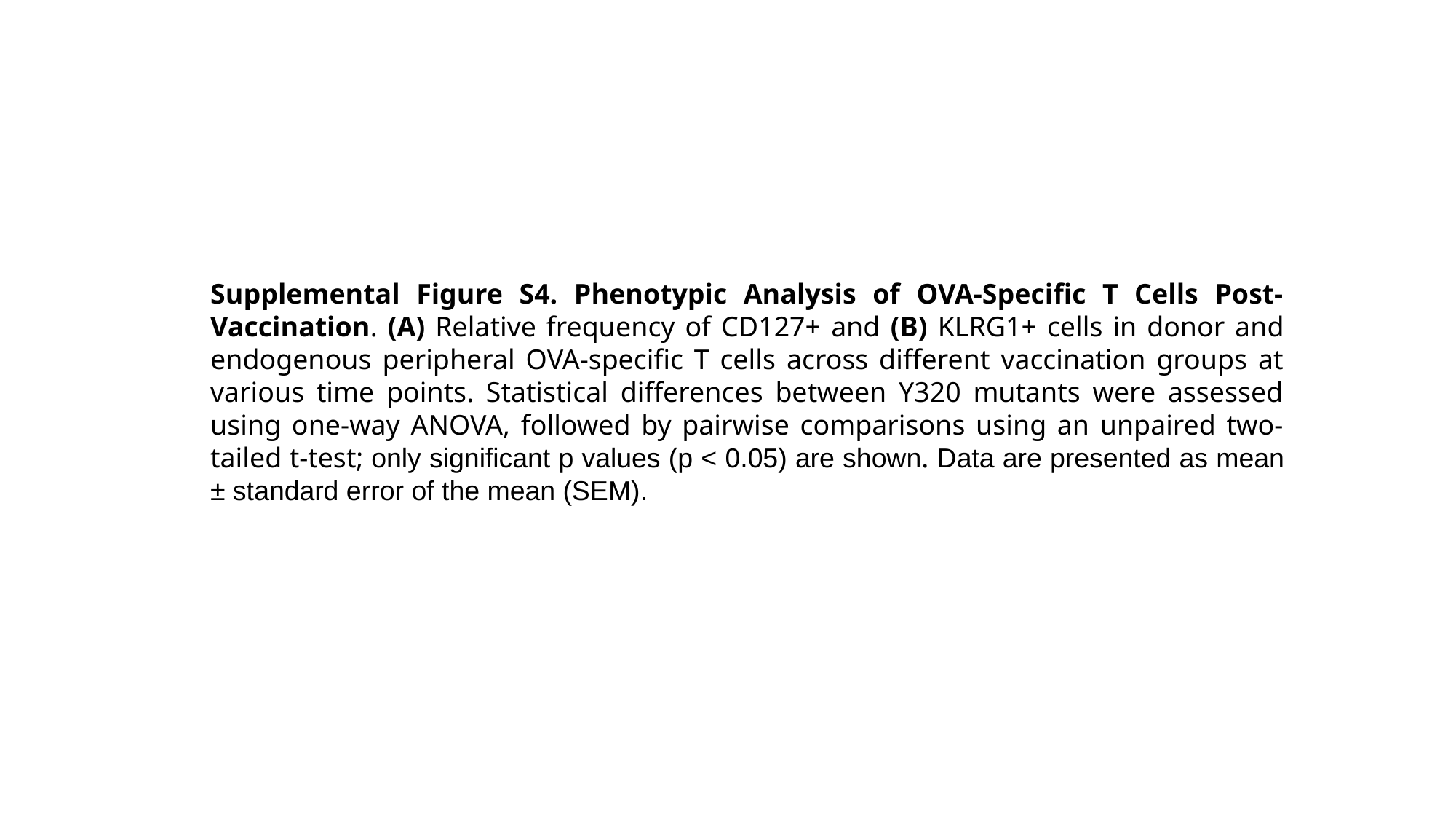

Supplemental Figure S4. Phenotypic Analysis of OVA-Specific T Cells Post-Vaccination. (A) Relative frequency of CD127+ and (B) KLRG1+ cells in donor and endogenous peripheral OVA-specific T cells across different vaccination groups at various time points. Statistical differences between Y320 mutants were assessed using one-way ANOVA, followed by pairwise comparisons using an unpaired two-tailed t-test; only significant p values (p < 0.05) are shown. Data are presented as mean ± standard error of the mean (SEM).

## Slide 9
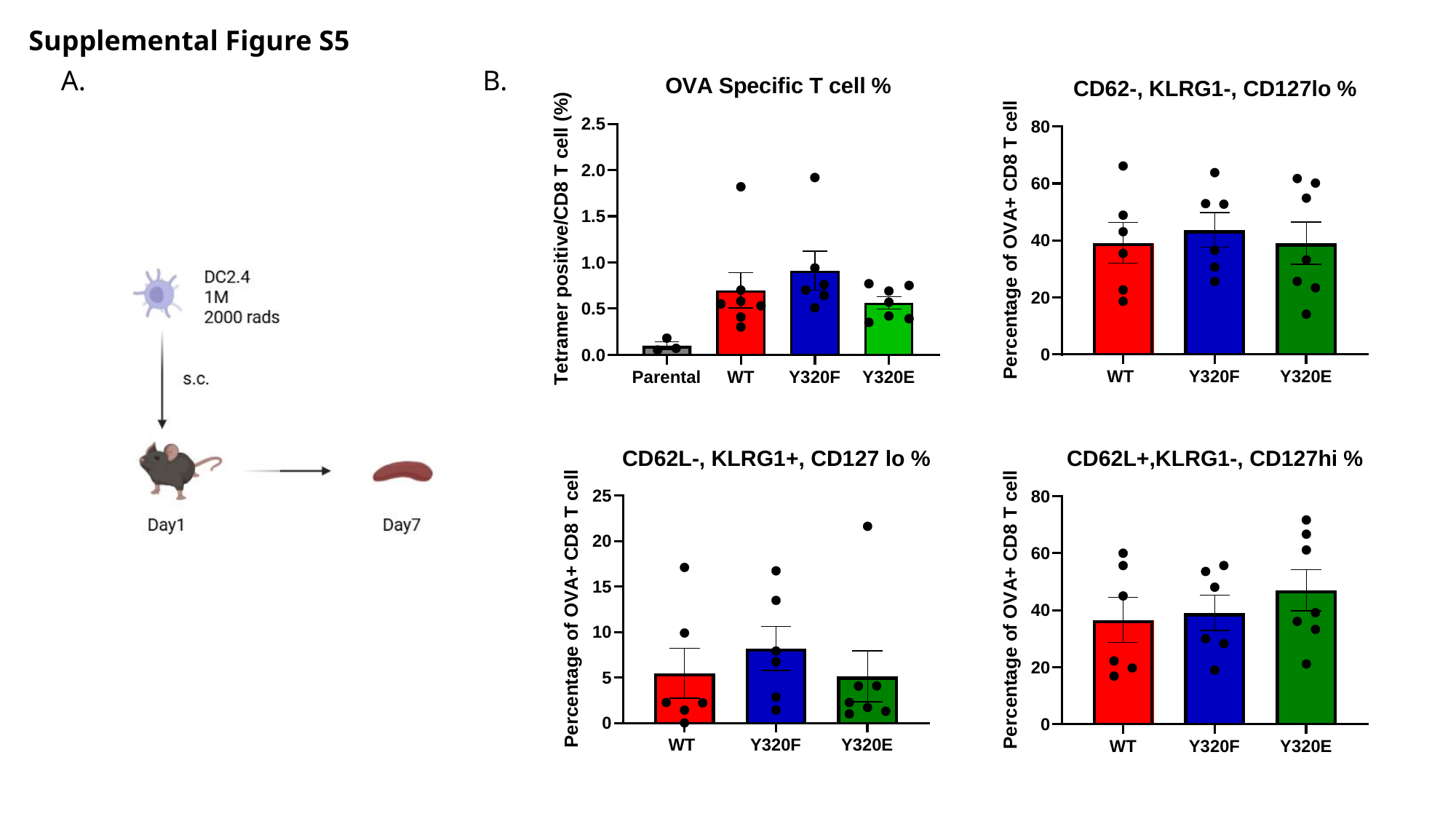

Supplemental Figure S5
A.
B.

## Slide 10
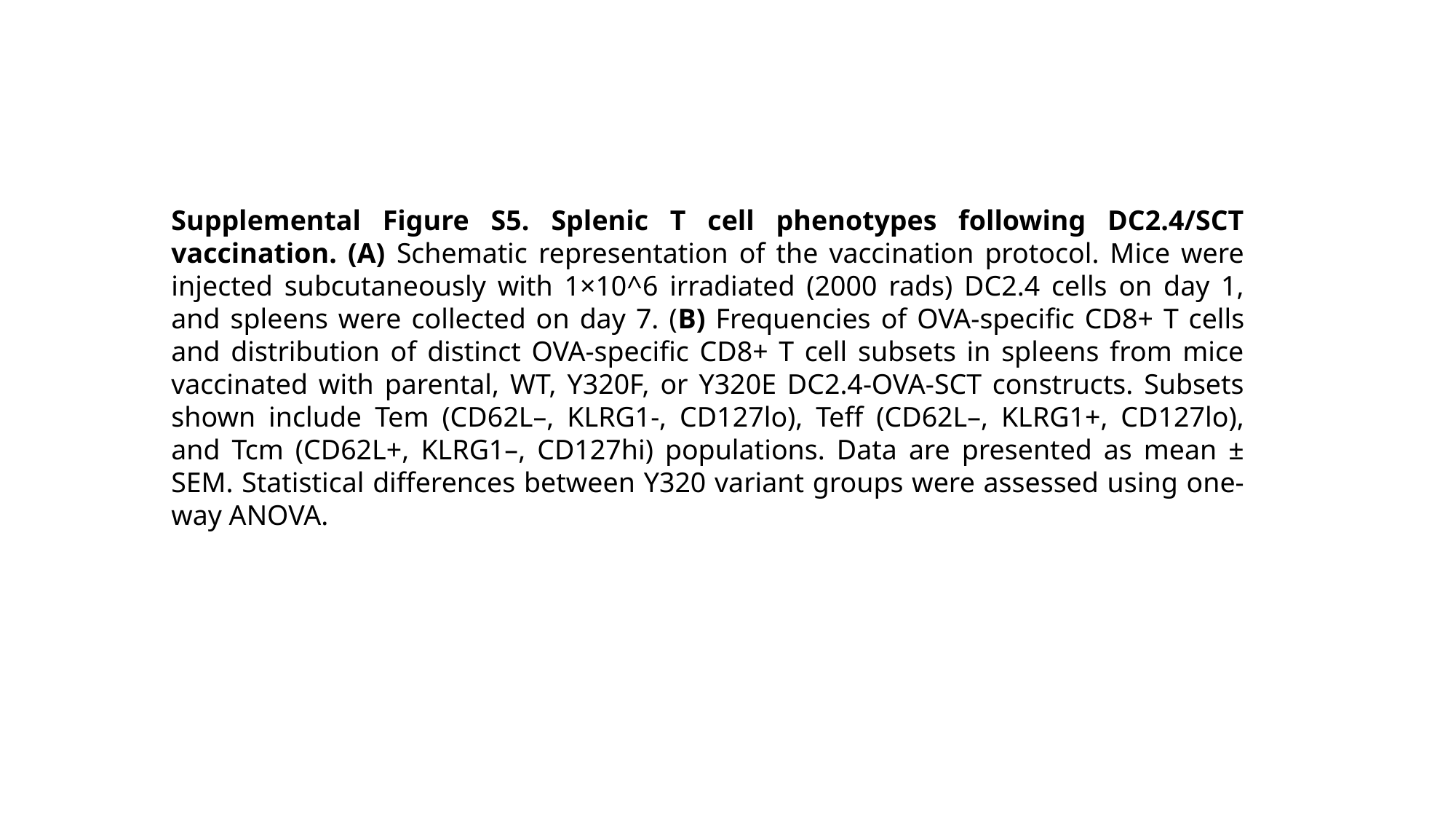

Supplemental Figure S5. Splenic T cell phenotypes following DC2.4/SCT vaccination. (A) Schematic representation of the vaccination protocol. Mice were injected subcutaneously with 1×10^6 irradiated (2000 rads) DC2.4 cells on day 1, and spleens were collected on day 7. (B) Frequencies of OVA-specific CD8+ T cells and distribution of distinct OVA-specific CD8+ T cell subsets in spleens from mice vaccinated with parental, WT, Y320F, or Y320E DC2.4-OVA-SCT constructs. Subsets shown include Tem (CD62L–, KLRG1-, CD127lo), Teff (CD62L–, KLRG1+, CD127lo), and Tcm (CD62L+, KLRG1–, CD127hi) populations. Data are presented as mean ± SEM. Statistical differences between Y320 variant groups were assessed using one-way ANOVA.

## Slide 11
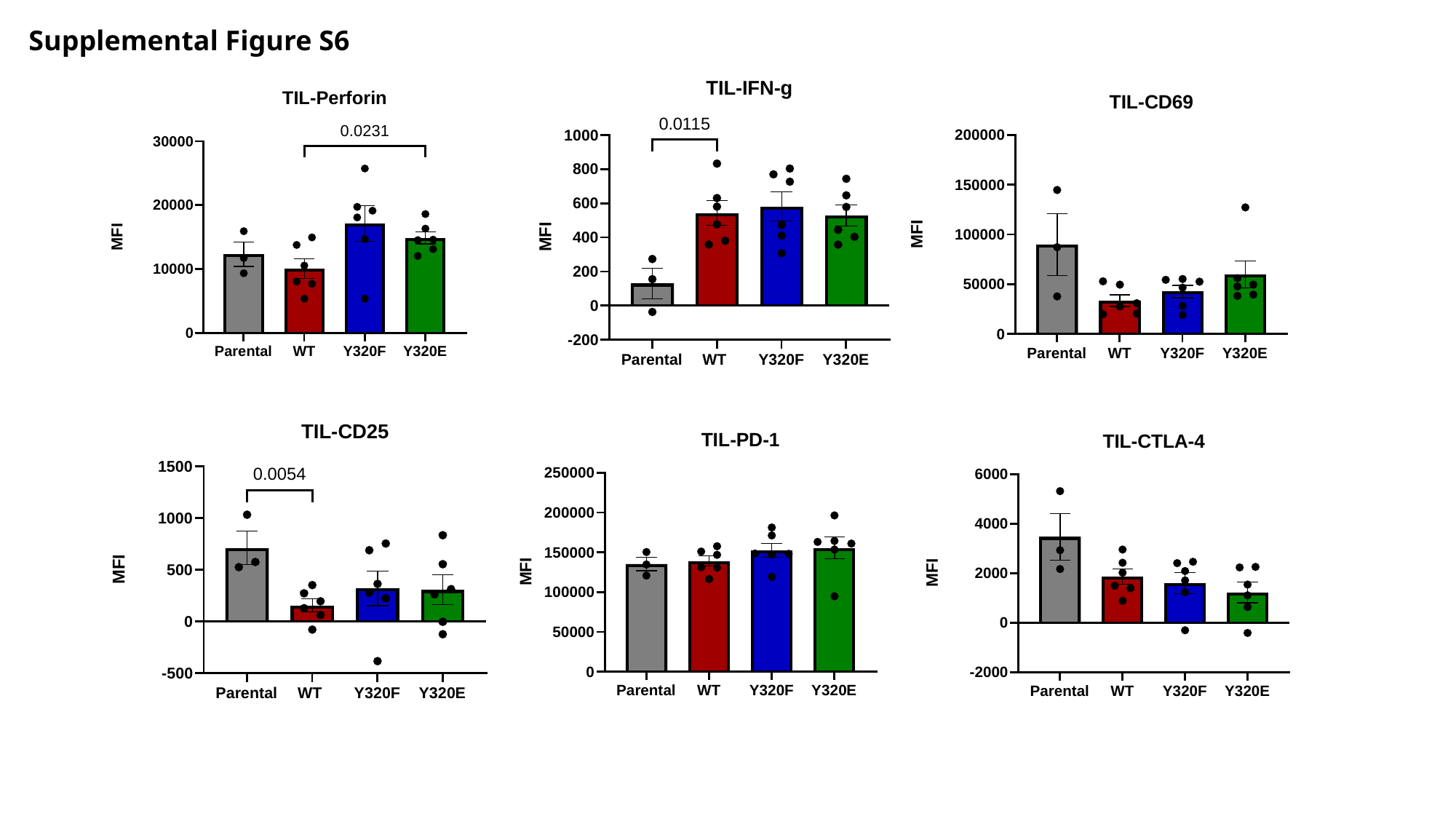

Supplemental Figure S6

## Slide 12
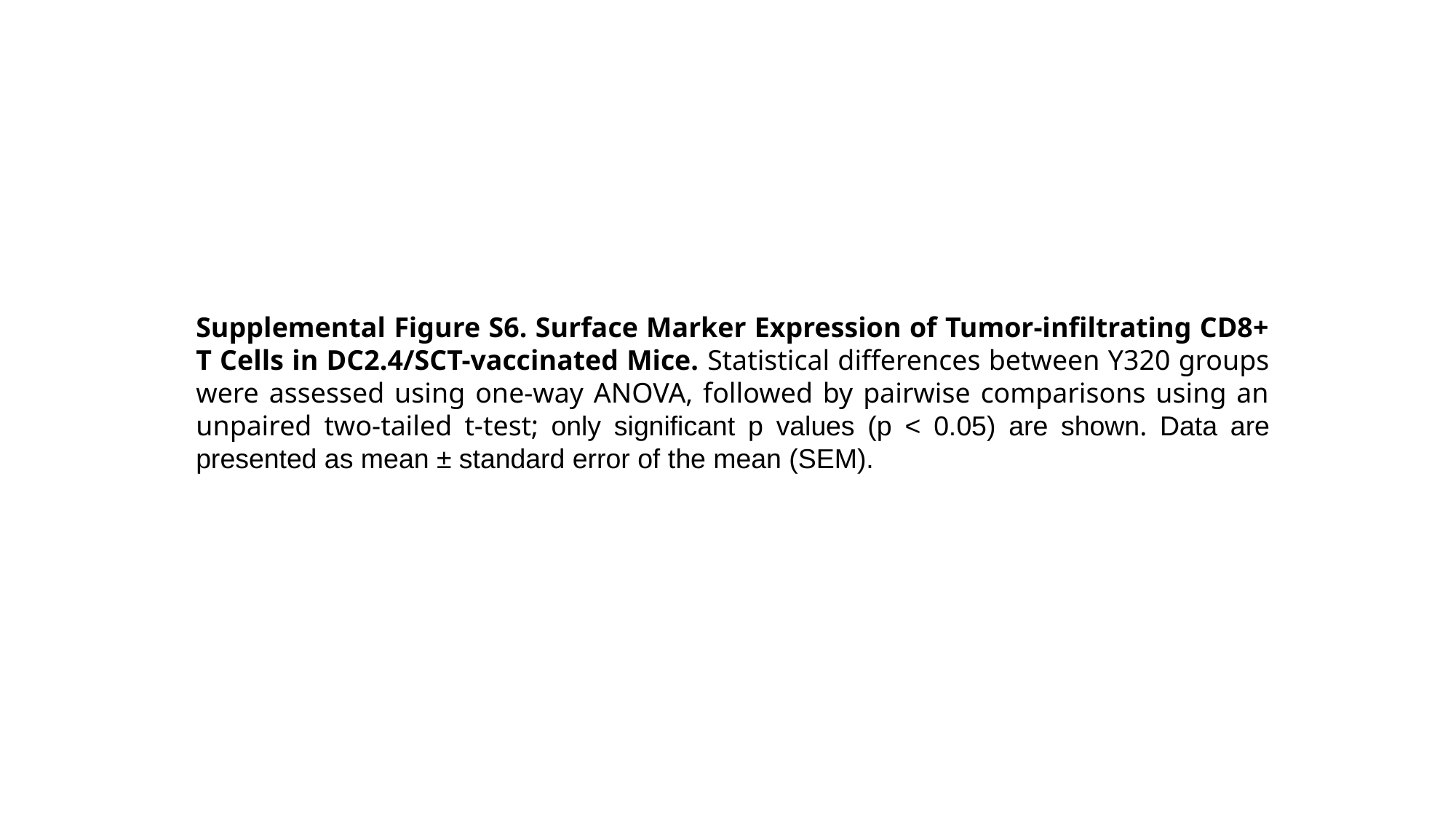

Supplemental Figure S6. Surface Marker Expression of Tumor-infiltrating CD8+ T Cells in DC2.4/SCT-vaccinated Mice. Statistical differences between Y320 groups were assessed using one-way ANOVA, followed by pairwise comparisons using an unpaired two-tailed t-test; only significant p values (p < 0.05) are shown. Data are presented as mean ± standard error of the mean (SEM).
